# Supplementary material for: Effects of PCSK9 inhibitors on coronary microcirculation, inflammation and cardiac function in patients with CHD after PCI: a protocol for systematic review and meta-analysis
Source: BMJ Open. 2023 Sep 18;13(9):e074067. doi: 10.1136/bmjopen-2023-074067 (PMC10510950; doi:10.1136/bmjopen-2023-074067)
Supplement: Supplementary data [file bmjopen-2023-074067supp001.pdf]

Table 1 Search strategy for PubMed

PCSK9 inhibitors [MeSH]

1. (proprotein convertase subtilisin/kexin type 9 monoclonal antibody) OR (PCSK9 inhibitor) OR (evolocumab) OR (alirocumab) OR (Praluent) OR (Repatha) [Title/Abstract]

Inflammation [MeSH]

2. (Inflammation) OR (Inflammatory response) OR (Inflammation reaction)

Randomized controlled trials [MeSH]

3. (RCT) OR (RCTs) OR (Randomized controlled trials) OR (Randomized controlled trial) [Title/Abstract]

Coronary heart disease[MeSH]

4. (Coronary artery disease) OR (Arteriosclerotic heart disease) OR (CAD) OR (CHD) OR (acute coronary syndrome) OR (chronic coronary syndrome) OR (ACS) OR (CCS)

5. 1 and 2 and 3 and 4

Table 2 Search strategy for EMBASE

PCSK9 inhibitors [MeSH]

1. (proprotein convertase subtilisin/kexin type 9 monoclonal antibody) OR (PCSK9 inhibitor) OR (evolocumab) OR (alirocumab) OR (Praluent) OR (Repatha) [Title/Abstract]

Inflammation [MeSH]

2. (Inflammation) OR (Inflammatory response) OR (Inflammation reaction)

Randomized controlled trials [MeSH]

3. (RCT) OR (RCTs) OR (Randomized controlled trials) OR (Randomized controlled trial) [Title/Abstract]

Coronary heart disease[MeSH]

4. (Coronary artery disease) OR (Arteriosclerotic heart disease) OR (CAD) OR (CHD) OR (acute coronary syndrome) OR (chronic coronary syndrome) OR (ACS) OR (CCS)

5. 1 and 2 and 3 and 4

Table 3 Search strategy for Web of Science

PCSK9 inhibitors [MeSH]

1. (proprotein convertase subtilisin/kexin type 9 monoclonal antibody) OR (PCSK9 inhibitor) OR (evolocumab) OR (alirocumab) OR (Praluent) OR (Repatha) [Title/Abstract]

Inflammation [MeSH]

2. (Inflammation) OR (Inflammatory response) OR (Inflammation reaction)

Randomized controlled trials [MeSH]

3. (RCT) OR (RCTs) OR (Randomized controlled trials) OR

(Randomized controlled trial) [Title/Abstract]

Coronary heart disease[MeSH]

4. (Coronary artery disease) OR (Arteriosclerotic heart disease) OR (CAD) OR (CHD) OR (acute coronary syndrome) OR (chronic coronary syndrome) OR (ACS) OR (CCS)

5. 1 and 2 and 3 and 4

Table 4 Search strategy for CNKI

PCSK9 抑制剂

1.(前蛋白转化酶枯草杆菌蛋白酶/可辛 9 型单克隆抗体) 或 (PCSK9 抑制剂) 或 (依洛尤单抗) 或 (阿利西尤单抗) 或 (波立达) 或 (瑞百安) [主题]

2. (炎症) 或 (炎症反应)

随机对照试验

3. (随机对照) 或 (随机对照试验) [主题]

冠状动脉粥样硬化性心脏病

4. (冠状动脉粥样硬化性心脏病) 或 (冠心病) 或 (急性冠脉综合征) 或 (慢性冠脉综合征) 或 (急性心肌梗死) 或 (ST 段抬高型心肌梗死) 或 (心绞痛) 或 (稳定性心绞痛) 或 (不稳定性心绞痛)

5. 1 和 2 和 3 和 4

Table 5 Search strategy for Wanfang

PCSK9 抑制剂
1.(前蛋白转化酶枯草杆菌蛋白酶/可辛 9 型单克隆抗体) 或 (PCSK9 抑制剂) 或 (依洛尤单抗) 或 (阿利西尤单抗) 或 (波立达) 或 (瑞百安) [主题]
2. (炎症) 或 (炎症反应)
随机对照试验
3. (随机对照) 或 (随机对照试验) [主题]
冠状动脉粥样硬化性心脏病
4. (冠状动脉粥样硬化性心脏病) 或 (冠心病) 或 (急性冠脉综合征) 或 (慢性冠脉综合征) 或 (急性心肌梗死) 或 (ST 段抬高型心肌梗死) 或 (心绞痛) 或 (稳定性心绞痛) 或 (不稳定性心绞痛)
5. 1 和 2 和 3 和 4

Table 6 Search strategy for VIP database

PCSK9 抑制剂
1.(前蛋白转化酶枯草杆菌蛋白酶/可辛 9 型单克隆抗体) 或 (PCSK9 抑制剂) 或 (依洛尤单抗) 或 (阿利西尤单抗) 或 (波立达) 或 (瑞百安) [主题]
2. (炎症) 或 (炎症反应)
随机对照试验
3. (随机对照) 或 (随机对照试验) [主题]

冠状动脉粥样硬化性心脏病

4. (冠状动脉粥样硬化性心脏病) 或 (冠心病) 或 (急性冠脉综合征) 或 (慢性冠脉综合征) 或 (急性心肌梗死) 或 (ST 段抬高型心肌梗死) 或 (心绞痛) 或 (稳定性心绞痛) 或 (不稳定性心绞痛)

5. 1 和 2 和 3 和 4
