# Supplementary material for: Effects of PCSK9 inhibitors on coronary microcirculation, inflammation and cardiac function in patients with CHD after PCI: a protocol for systematic review and meta-analysis
Source: BMJ Open. 2023 Sep 18;13(9):e074067. doi: 10.1136/bmjopen-2023-074067 (PMC10510950; doi:10.1136/bmjopen-2023-074067)
Supplement: Author's manuscript [file bmjopen-2023-074067.draft_revisions.pdf]

BMJ Open is committed to open peer review. As part of this commitment we make the peer review history of every article we publish publicly available.

When an article is published we post the peer reviewers' comments and the authors' responses online. We also post the versions of the paper that were used during peer review. These are the versions that the peer review comments apply to.

The versions of the paper that follow are the versions that were submitted during the peer review process. They are not the versions of record or the final published versions. They should not be cited or distributed as the published version of this manuscript.

BMJ Open is an open access journal and the full, final, typeset and author-corrected version of record of the manuscript is available on our site with no access controls, subscription charges or pay-per-view fees (http://bmjopen.bmj.com).

If you have any questions on BMJ Open's open peer review process please email
info.bmjopen@bmj.com

BMJ Open

Effects of PCSK9 inhibitors on coronary microcirculation, inflammation, and cardiac function in patients with CHD after PCI: A protocol for systematic review and meta-analysis.

Journal:	*BMJ Open*
Manuscript ID	bmjopen-2023-074067
Article Type:	Protocol
Date Submitted by the Author:	28-Mar-2023
Complete List of Authors:	YE, Xuejiao; China Academy of Chinese Medical Sciences, Guang’anmen Hospital<br>Wang, Shihan ; China Academy of Chinese Medical Sciences, Department of Cardiology<br>Wu, Qian; China Academy of Chinese Medical Sciences Guang'anmen Hospital<br>LV, Yanfei; Shanghai Qianhe Technology Co LTD<br>Lv, Qianyu; China Academy of Chinese Medical Sciences Guang'anmen Hospital<br>Li, Junjia; China Academy of Chinese Medical Sciences Guang'anmen Hospital<br>Li, Lanlan; China Academy of Chinese Medical Sciences Guang'anmen Hospital<br>Yang, Yingtian; Beijing University of Chinese Medicine,<br>LIU, Xiao’an; Capital Medical University
Keywords:	Coronary heart disease < CARDIOLOGY, Pharmacology < TROPICAL MEDICINE, Cardiac surgery < SURGERY

SCHOLARONE™
Manuscripts

Effects of PCSK9 inhibitors on coronary microcirculation, inflammation, and cardiac function in patients with CHD after PCI: A protocol for systematic review and meta-analysis.

Xuejiao YE[1], Shihan WANG[1*], Qian WU[1], Yanfei LV[3], Qianyu LV[1], Junjia LI[1], Lanlan LI[1], Yingtian YANG[1], Xiao'an LIU[2]

1 Guang'anmen Hospital, Chinese Academy of Chinese Medical Sciences, Beijing, China, 2 Capital University of Medical, Beijing, China, 3 Shanghai Qianhe Technology Co., LTD

*CORRESPONDENCE : Shihan Wang.
Email address : wangshihan91@126.com.
Affiliations : Guang'anmen Hospital, Chinese Academy of Chinese Medical Sciences, Beijing, China.

Abstract:

Introduction: Coronary heart disease(CHD) is one of the common diseases that seriously endanger human health. Percutaneous coronary intervention(PCI) is indispensable treatment for CHD. Most patients may experience coronary microcirculation disorders and thrombosis after surgery, which directly affects the efficacy of PCI and the prognosis of patients. Preprotein converting enzyme subtilisin/Kexin 9(PCSK9) inhibitors is a new type of pleiotropy lipid lowering drug with dual anti-inflammatory and lipid lowering effects. We conducted this protocol to systematically evaluate the effects of PCSK9 inhibitors on coronary microcirculation, inflammation and cardiac function in patients with

For peer review only - http://bmjopen.bmj.com/site/about/guidelines.xhtml

CHD after PCI, and to provide high-quality evidence-based evidence for the clinical application of PCSK9 inhibitors.

Methods and analysis: We will conduct and report the meta-analysis strictly based on the 2020 Preferred Reporting Items for Systematic Reviews and Meta-analyses Protocols Guidelines (PRISMA-P). This protocol had been registered in PROSPERO (Registration number CRD42022346189). We will search PubMed, EMBASE, Web of Science, the Cochrane library and four Chinese databases(CNKI, CBM, WANFANG, VIP database) according to preset search strategies, without language and publication time restrictions. We will cooperate with manual retrieval and will check the reference lists of identified relevant RCTs. Google Scholar will be searched to identify any grey literature. The literature finally included must meet the established inclusion criteria. The title, abstract and full text will be independently extracted by two reviewers and differences will be resolved through discussion or the involvement of a third reviewer. The extracted data will be analyzed with Review Manager 5.3. The Cochrane Risk of Bias Tool will be used to evaluate the risk of bias. Publication bias will be assessed by funnel plots. Heterogeneity will be assessed by the I^2 test and subgroup analyses will be used to further investigate the potential sources of

For peer review only - http://bmjopen.bmj.com/site/about/guidelines.xhtml

heterogeneity. The quality of literature will be assessed by GRADE score.

PROSPERO registration number: CRD42022346189

Keywords: Efficacy, PCSK9 inhibitors, coronary heart disease, post-operation of PCI, coronary microcirculation, inflammation, cardiac function.

1. Introduction

Coronary heart disease(CHD) is a cardiovascular disease caused by coronary atherosclerosis, which causes stenosis or occlusion of vascular lumen and leads to myocardial ischemia, hypoxia or necrosis[1]. Percutaneous coronary intervention(PCI), coronary artery bypass grafting(CABG) and medical treatment(MT) are three effective treatments for CHD, especially PCI is currently the major treatment of coronary artery multivessel lesions, has been used more widely in clinic[2]. With higher vascular patency rate and lower long-term vascular reconstruction risk, it can effectively improve myocardial blood flow perfusion by dredging the narrow or occluded coronary artery lumen[3]. Accompanying the obvious benefit, there are certain risks. The interaction of various factors in the process of surgery and after surgery has broken the balance of the internal environment, leading to a significant increase in the risk of major

For peer review only - http://bmjopen.bmj.com/site/about/guidelines.xhtml

cardiovascular adverse events(MACEs)[4].

Effective improvement of coronary microcirculation after PCI is of great significance for the prognosis of patients with CHD. Lipid management is one of the routine interventions after PCI, and is the key to ensuring coronary microcirculation patency and preventing stent thrombosis. Statins are the cornerstone of current lipid lowering therapy. Their cardiovascular protective effect was linearly correlated with the magnitude of low-density lipoprotein cholesterol (LDL-C) reduction. However, due to “the 6% effect”, doubling the dose of statins can only reduce LDL-C level by another 6%[5] and the existence of adverse reactions such as rhabdomyolysis and liver dysfunction limit the application of the drugs. PCSK9 inhibitors is one of the most promising non-statins lipid lowering drugs in the post statins era. They increase LDL-C clearance by inhibiting the binding of PCSK9 and low-density lipoprotein receptor(LDL-R) and have a stronger LDL-C lowering effect than statins. Studies have shown that PCSK9 inhibitors can reduce the LDL-C level by 50%~70% either alone or in combination with statins and also lowering other blood lipid indicators[6]. ODYSSEY-LONG TERM[7] test shows that PCSK9 inhibitors combined with statins can reduce the risk of MACEs in cardiovascular high-risk patients by about 50%. This

For peer review only - http://bmjopen.bmj.com/site/about/guidelines.xhtml

provides a theoretical basis for the safety for the application of PCSK9 inhibitors. Compared with statins, the better effect of lowering lipid and fewer side effects making PCSK9 inhibitors can be widely used in clinical practice[8]. Recent studies found that[9] in addition to lowering blood lipids and stabilizing plaque, they also play an anti-inflammatory role and can effectively reduce the risk of MACEs after PCI[10].

Some studies suggest that surgical procedures can expose coagulative tissue, increase blood lipid levels, and aggravate inflammation. Stent implantation can also induce platelet activation and aggregation, leading to in-stent thrombosis, both of which can increase the risk of MACEs[11]. Bruins and colleagues[12] proved the existence of inflammatory reactions by measuring indicators such as C-reactive protein(CRP) and Interleukin-6(IL-6) in the early postoperative period. BAKTASHIAN M et al. found that the increase of serum inflammatory factor hs-CRP is closely related to in-stent restenosis.[13] Hs-CRP induced activated immune response can adversely affect endothelial function, leading to unstable progression of immune dependent atherosclerotic plaque, and greatly increasing the risk of cardiovascular adverse events[14]. Some clinical studies also indicate that abnormal activation of inflammatory reactions is closely related

For peer review only - http://bmjopen.bmj.com/site/about/guidelines.xhtml

to the occurrence of adverse cardiovascular events after PCI[15, 16].

Considering the cardioprotective effect of PCSK9 inhibitors, and there is currently no literature evaluate its effects after PCI. Thus we propose this protocol to systematically evaluate the effects of PCSK9 inhibitors on coronary microcirculation, inflammation, and cardiac function in patients with CHD after PCI, and to provide high-quality evidence-based evidence for the clinical application.

2. Objective

① The primary objectives are blood lipid indicators, such as LDL-C, total cholesterol (TC), triglyceride (TG), high density lipoprotein cholesterol (HDL-C) and serum inflammatory factors, such as CRP/high-sensitivity CRP(hs-CRP), IL-6, tumor necrosis factor-α (TNF-α)、matrix metalloproteinase-9(MMP-9).

② The secondary objectives are coronary blood flow classification and indicators of cardiac function, such as left atrial diameter (LAD), left ventricular ejection fraction (LVEF), E/A and the incidence of MACEs.

3. Methods and analysis

3.1 Protocol registration

For peer review only - http://bmjopen.bmj.com/site/about/guidelines.xhtml

This protocol is conceived following the 2020 preferred reporting items for systematic review and meta-analysis protocols (PRISMA-P) statement[17] and has been registered on the International Prospective Register of Systematic Reviews(PROSPERO) network. Registration number is CRD4202346189.

3.2 Date sources and search strategies

For this article, we will search PubMed, EMBASE, Web of Science, the Cochrane Library and four Chinese databases(CNKI, CBM, WANFANG, VIP database). Google Scholar will be searched to identify any grey literature. We will cooperate with manual retrieval and will check the reference lists to identified relevant randomized controlled trials(RCTs).

The search term is a combination of free words and subject terms, including: postoperative PCI, PCSK9 inhibitors, PCSK9, inflammatory, etc. The search terms were connected by Boolean operators. We will provide a detailed search strategy for PubMed, as shown in Table 1. The terms will be modified as necessary for other databases. We will not apply any language and date restrictions. Duplicate literature will be automatically removed by EndNote (version X9, Clarivate Analytics), and the remaining duplicate

For peer review only - http://bmjopen.bmj.com/site/about/guidelines.xhtml

literature will be manually removed by comparing authors, title and publication date. All literature that meets the inclusion criteria will be further screened and read the full text to extract the required data, and excluded literature will be recorded with the reason. This process will be conducted independently by two reviewers, and any problems and differences will be resolved through mutual discussion or discussion with a third reviewer. In line with the PRISMA-P guidelines, a flow diagram will summarize the entire study selection process (Fig 1).

Table 1 Search strategy for PubMed

**Search strategy**
PCSK9 inhibitors
1. PCSK9 inhibitors
2. (PCSK9) OR (proprotein convertase subtilisin/kexin type 9 monoclonal antibody) OR (proprotein convertase subtilisin/kexin type 9 inhibitor) OR (PCSK9 inhibitor) OR (PCSK9 antibody) OR (evolocumab) OR (alirocumab) OR (bocucizumab) OR (RG7652) OR (AMG145) OR (REGN727) OR (RN 316) OR (SAR236553) OR (PF-04950615) [Title/Abstract]
3. 1 or 2
4. (inflammatory) OR (inflammatory response) OR (inflammation) OR (inflammation reaction)

For peer review only

For peer review only - http://bmjopen.bmj.com/site/about/guidelines.xhtml

RCTs
5. Randomized controlled trials [MeSH]
6. (RCT) OR (RCTs) OR (Randomized controlled trials) OR (Randomized controlled trial) [Title/Abstract]
7. 5 or 6
8. 3 and 4 and 7

3.3 Eligibility criteria for study selection

3.3.1 Study design

RCTs comparing the effects of PCSK9 inhibitors on coronary microcirculation, inflammation, and cardiac function in patients with CHD after PCI will be included, without time and language restrictions. Non-randomized controlled trials, observational studies, animal experiments, case report, reviews and conference abstracts will be excluded.

3.3.2 Participants

Inclusion criteria:

① Male or female patients who meet the diagnostic criteria for CHD aged≥18 years and ≤85 years[18].

② Completed PCI treatment within 12 hours of onset

For peer review only - http://bmjopen.bmj.com/site/about/guidelines.xhtml

③ Patients who are able to tolerant and willing to receive basic PCSK9 inhibitors or statins or ezetimibe lipid-lowering treatment.

Exclusion criteria:

① Intolerant to statins, ezetimibe and PCSK9 inhibitors.

② Severe liver and kidney dysfunction.

③ Pregnant or lactating women.

④ Malignant tumor or blood system disease.

⑤ History of severe allergic reactions to lipid-lowering drugs.

⑥ Unsuitable participants for any other reasons, as judged by the reviewers.

3.3.3 Types of interventions

Any RCTs using PCSK9 inhibitors before PCI will be included. PCSK9 inhibitors include evolocumab or alirocumab, for 140mg/2weeks or 420mg/4weeks. Interventions will be included but not limited to the following:

① PCSK9 inhibitors VS placebo;

② PCSK9 inhibitors VS statins;

③ PCSK9 inhibitors＋statins VS statins;

④ PCSK9 inhibitors VS statins and ezetimibe;

⑤ PCSK9 inhibitors ＋statins VS statins and ezetimibe;

⑥ PCSK9 inhibitors ＋statins VS ezetimibe＋statins.

For peer review only - http://bmjopen.bmj.com/site/about/guidelines.xhtml

3.3.4 Types of outcome measures Eligible studies should report at least one of the following outcomes.

Primary outcomes include:

① The primary objectives are blood lipid indicators, such as LDL-C, TC, TG, HDL-C and serum inflammatory factors, such as CRP/ hs-CRP, IL-6, TNF-α and MMP-9.

② The secondary objectives are coronary blood flow classification and indicators of cardiac function, such as LAD, LVEF, E/A and the incidence of MACEs(MACEs:including the recurrent of myocardial infarction, recurrent PCI/CABG, heart failure, malignant arrhythmia, ischemic stroke, Cardiogenic shock, Cardiac arrest, Cardiogenic death[19, 20])[21].

3.4 Data collection and analysis

3.4.1 Date extraction and management

After screening the literature based on inclusion and exclusion criteria, the self-made Microsoft Excel data extraction table will be used for data extraction of the meta-analysis, which presented in supplementary materials. This process will be performed by two reviewers independently and any disagreements will be resolved through mutual discussion or discussion with a third reviewer. The

For peer review only - http://bmjopen.bmj.com/site/about/guidelines.xhtml

following data will be extracted:

① Study identification: first author's name, title, year of publication.

② Study characteristics: study design (RCTs), duration of follow-up for clinical trials, number of cases per group.

③ Study population: sample size, mean or median age, gender composition ratio, proportion of patients with diabetes or hypertension, basic disease, basic medication.

④ Interventions: type and dose of administration, course of treatment, frequency of administration.

⑤ Primary outcomes: blood lipid indicators, such as LDL-C, TC, TG, HDL-C and serum inflammatory factors, such as CRP/hs-CRP, IL-6, TNF-α, and MMP-9.

⑥ Secondary outcomes: coronary blood flow classification and indicators of cardiac function, such as LAD, LVEF, E/A and the incidence of MACEs.

3.4.2 Data synthesis and analysis

We will apply Review Manager 5.3 provided by the Cochrane Collaboration(http://www-cochrane-org.vpn1.hactcm.edu.cn/) to analyze the obtained data[22]. For continuous variables, mean value (MD) and 95% confidence intervals(CI) will be used for data analysis. For dichotomous variables, odds ratio(OR) and 95% CI will be

For peer review only - http://bmjopen.bmj.com/site/about/guidelines.xhtml

applied. $P \geq 0.05$ indicates no statistical significance, and $P < 0.05$ indicates statistical significance. The heterogeneity of the literature will be evaluated by the I^2 test. If $P \geq 0.1$ and $I^2 \leq 50\%$, there is no statistical heterogeneity, and the fixed effect model will be used. If $P < 0.1$ and $I^2 > 50\%$, indicates statistical heterogeneity exists, and random effect model will be used.

3.5 Dealing with missing data

For incomplete or missing data, we will contact the corresponding author to request additional information. Otherwise, related literature will be excluded.

3.6 Risk assessment of included literature

The Cochrane Collaboration's risk of bias assessment tool(http://www-cochrane-org.vpn1.hactcm.edu.cn/) will be applied to independently assess the bias risk of each included literature[23]. We will assess the risk of bias from six domains: performance bias (blinding of participants and personnel), selection bias (random sequence generation, allocation concealment), detection bias (blinding of outcome assessment), attrition bias (incomplete outcome data), reporting bias (selective reporting) and other potential biases. The quality of assessment is divided into three grades: low risk of bias,

For peer review only

For peer review only - http://bmjopen.bmj.com/site/about/guidelines.xhtml

some concerns, high risk of bias. For duplicate literature, we will select only the original version. Any problems and differences will be resolved by discussion between the two reviewers, and if necessary, seeking help from a third reviewer. Review Manager 5.3 software will be used to plot the results of the risk assessment.

3.7 Publication of bias

We will use the funnel plot to visually assess publication bias if the number of included literature is more than 10. Symmetry on both sides of the funnel diagram indicates that there is no publication bias, while asymmetric distribution on both sides indicates possible publication bias. If the number of literature is less than 10, the Egger test will be used for statistical analysis. $P < 0.05$ indicates the existence of publication bias, while $P \geqslant 0.05$ indicates the absence of publication bias.

3.8 Data analysis

3.8.1 Assessment of heterogeneity

We will use I^2 statistic to assess the heterogeneity of included literature, as the criteria: $I^2 \leqslant 50\%$ indicates low heterogeneity, while $I^2 > 50\%$ indicates high heterogeneity.

For peer review only - http://bmjopen.bmj.com/site/about/guidelines.xhtml

3.8.2 Subgroup analysis and the sources of heterogeneity

When heterogeneity is evident, we will conduct subgroup analysis to investigate the source of heterogeneity:

① Age.

② Race.

③ Proportion of patients with hypertension or diabetes.

④ Types and doses of interventions.

3.8.3 Sensitivity analyses

We will use a one-by-one exclusion method for sensitivity analysis to evaluate the robustness of the study results. In the process of sensitivity analysis, multiple uncertainties need to be considered, such as small sample size, missing data and methodological quality. Repetitive analysis is required after excluding literature with low methodological quality.

3.8.4 Quality evaluation of included literature

We well use GRADE score[24] to evaluate the quality of the included literature, and each literature will be stratified according to high-level evidence, intermediate evidence, low-level evidence and extremely low-level evidence.

For peer review only - http://bmjopen.bmj.com/site/about/guidelines.xhtml

4. Discussion

With the sharp rise in the prevalence of hyperlipidemia, the morbidity and mortality of CHD further increased. Atherosclerosis is the basis of CHD, and dyslipidemia is the core factor of atherosclerosis. Effective control of lipid levels is becoming the key to slowing down the progression of atherosclerosis and preventing the occurrence of CHD[25]. Several clinical studies published by the 2023 Annual Scientific Conference of the American College of Cardiology (ACC) and the World Conference of the Cardiology Society (WCC) show that inflammation and hyperlipidemia are the key to atherothrombotic disease[26]. Clinical studies have also shown that although PCI can quickly clear coronary artery stenosis or occlusion in patients with CHD and correct the blood supply to the heart, a large amount of inflammatory reactions are activated, leading to an increased risk of postoperative MACEs in patients. Intensive lipid-lowering therapy, especially lowering serum LDL-C levels, can effectively reduce the risk of postoperative MACEs, which is significant for improving the prognosis of patients with CHD[27]. Statins are currently the cornerstone of clinical lipid-lowering therapy and have anti-inflammatory effects. They can effectively reduce the risk of atherosclerotic cardiovascular disease (ASCVD) in patients. However, in clinical practice, we have found that the LDL-C level of most

For peer review only - http://bmjopen.bmj.com/site/about/guidelines.xhtml

patients after PCI does not meet the standard, which may be related to the "6% rule" of statins and some patients' intolerance to statins[28]. At the same time, among patients with ASCVD receiving intensive statins treatment, the residual inflammatory risk is an important risk factor for long-term cardiovascular events. Therefore, the combination of intensive lipid-lowering therapy and anti-inflammatory therapy will provide greater benefits for high-risk patients with ASCVD[29, 30].

PCSK9 inhibitors is a new type of non-statin lipid lowering drug that reduces LDL-R degradation and increases serum LDL-C clearance by inhibiting the binding of PCSK9 and LDL-R[31]. Studies have shown that PCSK9 inhibitors and statins have synergistic effects. Adding PCSK9 inhibitors Aliciumab or Evolocumab to intensive statins therapy can effectively improve the stability of intracoronary plaque and achieve greater cardiovascular benefits for patients with CHD[32, 33]. Inflammatory response of plaque is one of the important inducements for the occurrence and development of atherosclerosis. Intensive anti-inflammatory treatment is also one of the important intervention strategies after PCI[34]. PCSK9, as a secreted serine protease, is mainly synthesized and secreted by the liver[35]. It can directly participate in the inflammatory reaction process of AS by

For peer review only - http://bmjopen.bmj.com/site/about/guidelines.xhtml

influencing the concentration of LDL-C, HDL-C, TG, and lipoprotein a(Lp (a)) in the circulation[36], and can also promote the occurrence of plaque inflammation by increasing the infiltration and differentiation of monocytes[37]. Moreover, animal experiments show that PCSK9 inhibitors can play a role in regulating blood lipids by mediating inflammatory signaling pathways, inhibiting the progress of atherosclerosis and promoting plaque stability[38]. Therefore, in addition to its strong lipid lowering effect, PCSK9 inhibitors can alleviate inflammatory reactions and exert anti-inflammatory effects by reducing LDL-C levels, reducing LDL-R mediated platelet activation, and other ways[39, 40].

Exploring new targets for lipid lowering therapy has always been a research hotspot in the field of lipid lowering therapy. Recently, the emergence of a new oral PCSK9 inhibitor MK-0616 in clinical research has broken through the limitations of the original injection type, which can safely and effectively reduce the serum LDL-C level of patients, while also having a similar lowering effect on apolipoprotein B(Apo B)[41]. The oral dosage form greatly improves the patient's medication compliance and provided a new choice for lowering blood lipids for ASCVD patients. This therapeutic effect still needs to be verified in more clinical trials in the future. The new type

For peer review only - http://bmjopen.bmj.com/site/about/guidelines.xhtml

of oral non-statin lipid lowering drug Bempedoic Acid mainly inhibits liver cholesterol synthesis by inhibiting ATP citrate lyase (ACL) at the upstream level of HMG CoA[42]. Moreover, this drug can only be metabolized by the liver, effectively avoiding the common adverse reactions of statins such as rhabdomyolysis[26]. Recently published clinical research results indicate that Bempedoic Acid has a significant reduction in LDL-C, but also improves other blood lipid indicators to varying degrees[43]. It can also effectively reduce the occurrence of MACEs, providing a new treatment strategy for ASCVD patients with statin intolerance[26].

5. Strengths and limitations

This protocol is the latest systematic review and meta-analysis to evaluate the effects of PCSK9 inhibitors on coronary microcirculation, inflammation, and cardiac function in patients with CHD after PCI. Up to now, there is no systematic collation and sharing of relevant data on this issue. Compared with the previous systematic review and meta-analysis, we take into account a wider range of outcome indicators, including laboratory indicators and ultrasound indicators. However, there are still many limitations, such as the number of

For peer review only - http://bmjopen.bmj.com/site/about/guidelines.xhtml

studies that meet our screening criteria may be limited. The reliability and representativeness of research conclusions still require more clinical research data to confirm. We look forward to further research in the future to open up new ideas and methods for the treatment of inflammation and coronary microcirculation disorders after PCI.

6. Ethics statement

This study is a secondary study based on data from published literature, no special ethical approval is required.

7. Author contributions

XY and SW designed the study. QW，QL，JL created the tables and figures. XY and YL wrote the initial draft of the manuscript. YY and LL revised the manuscript. All authors contributed to the article and approved the final version of the manuscript.

8. Funding

This research is supported by Beijing Municipal Science & Technology Commission (Z191100006619025).

9. Conflict of interest

For peer review only - http://bmjopen.bmj.com/site/about/guidelines.xhtml

The authors declare that the research was conducted in the absence of any commercial or financial relationships that could be construed as a potential conflict of interest.

10. Supplementary material

The Supplementary Material for this self-made Microsoft Excel data extraction table can be found online at: Microsoft Excel 数据提取表.xlsx

References:

[1]A. K. Malakar, D. Choudhury, B. Halder, P. Paul, A. Uddin and S. Chakraborty, ***2019***. A review on coronary artery disease, its risk factors, and therapeutics[J]. *J Cell Physiol*. 234[10], 16812-16823

[2]M. P. Sá, P. E. Ferraz, R. R. Escobar, E. O. Nunes, A. M. Soares, F. B. Araújo e Sá, F. P. Vasconcelos and R. C. Lima, ***2013***. Five-year outcomes following PCI with DES versus CABG for unprotected LM coronary lesions: meta-analysis and meta-regression of 2914 patients[J]. *Rev Bras Cir Cardiovasc*. 28[1], 83-92

[3]C. M. Gibson, R. Mehran, C. Bode, J. Halperin, F. W. Verheugt, P. Wildgoose, M. Birmingham, J. Ianus, P. Burton, M. van Eickels, S. Korjian, Y. Daaboul, G. Y. Lip, M. Cohen, S. Husted, E. D. Peterson and K. A. Fox, ***2016***. Prevention of Bleeding in Patients with Atrial Fibrillation Undergoing PCI[J]. *N Engl J Med*. 375[25], 2423-2434

[4]林珑, 刘冠男, 高丽霓, 王吟春, 李晓东, 孔晓琳, 李晓 and 张辰浩, ***2020***. 经皮冠状动脉介入术后主要不良心脏事件危险因素研究进展[J]. *临床急诊杂志*. 21[11], 918-922

[5]P. M. Ridker, S. Mora and L. Rose, ***2016***. Percent reduction in LDL cholesterol following high-intensity statin therapy: potential implications for guidelines and for the prescription of emerging lipid-lowering agents[J]. *Eur Heart J*. 37[17], 1373-1379

[6]Z. Ding, N. V. K. Pothineni, A. Goel, T. F. Lüscher and J. L. Mehta, ***2020***. PCSK9 and inflammation: role of shear stress, pro-inflammatory cytokines, and LOX-1[J]. *Cardiovasc Res*. 116[5], 908-915

[7]J. G. Robinson, M. Farnier, M. Krempf, J. Bergeron, G. Luc, M. Averna, E. S. Stroes, G. Langslet, F. J. Raal, M. El Shahawy, M. J. Koren, N. E. Lepor, C. Lorenzato, R. Pordy, U. Chaudhari and J. J. Kastelein, ***2015***. Efficacy and safety of alirocumab in reducing lipids and cardiovascular events[J]. *N Engl J Med*. 372[16], 1489-1499

For peer review only

For peer review only - http://bmjopen.bmj.com/site/about/guidelines.xhtml

[8]廖玉华 and 魏晶晶, ***2016***. 他汀和 PCSK9 抑制剂调节 LDL-C 合成与清除代谢的临床荟萃分析[J]. *临床心血管病杂志*. 32[06], 537-540

[9]A. A. Momtazi-Borojeni, S. Sabouri-Rad, A. M. Gotto, M. Pirro, M. Banach, Z. Awan, G. E. Barreto and A. Sahebkar, ***2019***. PCSK9 and inflammation: a review of experimental and clinical evidence[J]. *Eur Heart J Cardiovasc Pharmacother*. 5[4], 237-245

[10]L. J. Qian, Y. Gao, Y. M. Zhang, M. Chu, J. Yao and D. Xu, ***2017***. Therapeutic efficacy and safety of PCSK9-monoclonal antibodies on familial hypercholesterolemia and statin-intolerant patients: A meta-analysis of 15 randomized controlled trials[J]. *Sci Rep*. 7[1], 238

[11]张都生，吴志勇，林锋，陈威，许锦鸿 and 郭延松, ***2020***. 老年冠心病患者 PCI 术后发生冠状动脉微循环损伤的影响因素[J]. *山东医药*. 60[33], 31-34

[12]P. Bruins, H. te Velthuis, A. P. Yazdanbakhsh, P. G. Jansen, F. W. van Hardevelt, E. M. de Beaumont, C. R. Wildevuur, L. Eijsman, A. Trouwborst and C. E. Hack, ***1997***. Activation of the complement system during and after cardiopulmonary bypass surgery: postsurgery activation involves C-reactive protein and is associated with postoperative arrhythmia[J]. *Circulation*. 96[10], 3542-3548

[13]M. Baktashian, S. Saffar Soflaei, N. Kosari, M. Salehi, A. Khosravi, M. Ahmadinejad, M. Moohebati, M. Ebrahimi, F. Rahmani, R. Khameneh-Bagheri, M. Ahmadi, F. Sadabadi, M. Tayefi, S. Bazhdanzadeh, G. A. Ferns, S. M. Hashemi, A. Pasdar and M. Ghayour-Mobarhan, ***2019***. Association of high level of hs-CRP with in-stent restenosis: A case-control study[J]. *Cardiovasc Revasc Med*. 20[7], 583-587

[14]T. Chen, J. L. Sun and J. Zhang, ***2020***. The relationship between fibrinogen-to-albumin ratio and in-stent restenosis in patients with coronary artery disease undergoing drug-eluting stenting[J]. *Coron Artery Dis*. 31[7], 586-589

[15]罗勤，李姣，周国忠 and 黄文军, ***2021***. NLR、PLR、NT-proBNP 联合预测急性冠脉综合征患者急诊 PCI 后新发房颤的价值[J]. *分子诊断与治疗杂志*. 13[12], 2051-2055

[16]B. S. Karam, A. Chavez-Moreno, W. Koh, J. G. Akar and F. G. Akar, ***2017***. Oxidative stress and inflammation as central mediators of atrial fibrillation in obesity and diabetes[J]. *Cardiovasc Diabetol*. 16[1], 120

[17]M. J. Page, J. E. McKenzie, P. M. Bossuyt, I. Boutron, T. C. Hoffmann, C. D. Mulrow, L. Shamseer, J. M. Tetzlaff, E. A. Akl, S. E. Brennan, R. Chou, J. Glanville, J. M. Grimshaw, A. Hróbjartsson, M. M. Lalu, T. Li, E. W. Loder, E. Mayo-Wilson, S. McDonald, L. A. McGuinness, L. A. Stewart, J. Thomas, A. C. Tricco, V. A. Welch, P. Whiting and D. Moher, ***2021***. The PRISMA 2020 statement: An updated guideline for reporting systematic reviews[J]. *PLoS Med*. 18[3], e1003583

[18]G. N. Levine, E. R. Bates, J. A. Bittl, R. G. Brindis, S. D. Fihn, L. A. Fleisher, C. B. Granger, R. A. Lange, M. J. Mack, L. Mauri, R. Mehran, D. Mukherjee, L. K. Newby, P. T. O'Gara, M. S. Sabatine, P. K. Smith and S. C. Smith, Jr., ***2016***. 2016 ACC/AHA Guideline Focused Update on Duration of Dual Antiplatelet Therapy in Patients With Coronary Artery Disease: A Report of the American College of Cardiology/American Heart Association Task Force on Clinical Practice Guidelines: An Update of the 2011 ACCF/AHA/SCAI Guideline for Percutaneous Coronary Intervention, 2011 ACCF/AHA Guideline for Coronary Artery Bypass Graft Surgery, 2012 ACC/AHA/ACP/AATS/PCNA/SCAI/STS Guideline for the Diagnosis and Management of Patients With Stable Ischemic Heart Disease, 2013 ACCF/AHA Guideline for the Management of ST-Elevation Myocardial Infarction, 2014 AHA/ACC Guideline for the Management of Patients With Non-ST-Elevation Acute Coronary Syndromes, and 2014 ACC/AHA Guideline on

For peer review only

For peer review only - http://bmjopen.bmj.com/site/about/guidelines.xhtml

Perioperative Cardiovascular Evaluation and Management of Patients Undergoing Noncardiac Surgery[J]. *Circulation*. 134[10], e123-155

[19]L. De Luca, A. Rubboli, L. Bolognese, M. Uguccioni, D. Lucci, S. Blengino, J. Campodonico, I. Meynet, S. M. Brach Prever, A. Di Lenarda, D. Gabrielli and M. M. Gulizia, ***2021***. Is percutaneous coronary intervention safe during uninterrupted direct oral anticoagulant therapy in patients with atrial fibrillation and acute coronary syndromes?[J]. *Open Heart*. 8[2],

[20]D. L. E. Villanueva, M. D. Tiongson, J. D. Ramos and E. J. Llanes, ***2020***. Monocyte to High-Density Lipoprotein Ratio (MHR) as a predictor of mortality and Major Adverse Cardiovascular Events (MACE) among ST Elevation Myocardial Infarction (STEMI) patients undergoing primary percutaneous coronary intervention: a meta-analysis[J]. *Lipids Health Dis*. 19[1], 55

[21]Y. L. Alex Chau, J. Won Yoo, H. Chuen Yuen, K. Bin Waleed, D. Chang, T. Liu, F. Z. Liu, G. Tse, S. Lee and K. Hou Christien Li, ***2021***. The impact of post-operative atrial fibrillation on outcomes in coronary artery bypass graft and combined procedures[J]. *J Geriatr Cardiol*. 18[5], 319-326

[22]M. Cumpston, T. Li, M. J. Page, J. Chandler, V. A. Welch, J. P. Higgins and J. Thomas, ***2019***. Updated guidance for trusted systematic reviews: a new edition of the Cochrane Handbook for Systematic Reviews of Interventions[J]. *Cochrane Database Syst Rev*. 10[Ed000142

[23]J. A. C. Sterne, J. Savović, M. J. Page, R. G. Elbers, N. S. Blencowe, I. Boutron, C. J. Cates, H. Y. Cheng, M. S. Corbett, S. M. Eldridge, J. R. Emberson, M. A. Hernán, S. Hopewell, A. Hróbjartsson, D. R. Junqueira, P. Jüni, J. J. Kirkham, T. Lasserson, T. Li, A. McAleenan, B. C. Reeves, S. Shepperd, I. Shrier, L. A. Stewart, K. Tilling, I. R. White, P. F. Whiting and J. P. T. Higgins, ***2019***. RoB 2: a revised tool for assessing risk of bias in randomised trials[J]. *Bmj*. 366[l4898

[24]G. H. Guyatt, A. D. Oxman, G. E. Vist, R. Kunz, Y. Falck-Ytter, P. Alonso-Coello and H. J. Schünemann, ***2008***. GRADE: an emerging consensus on rating quality of evidence and strength of recommendations[J]. *Bmj*. 336[7650], 924-926

[25]X. Zhang and D. Hu, ***2015***. [Comments for 2013 ACC/AHA Guideline on the Treatment of Blood Cholesterol to Reduce Atherosclerotic Cardiovascular Risk in Adults][J]. *Zhonghua Nei Ke Za Zhi*. 54[1], 9-12

[26]黄丹 and 廖玉华, ACC 2023 热点速递:降脂治疗与心血管保护[J]. *临床心血管病杂志*. 1-3

[27]E. P. Navarese, J. G. Robinson, M. Kowalewski, M. Kolodziejczak, F. Andreotti, K. Bliden, U. Tantry, J. Kubica, P. Raggi and P. A. Gurbel, ***2018***. Association Between Baseline LDL-C Level and Total and Cardiovascular Mortality After LDL-C Lowering: A Systematic Review and Meta-analysis[J]. *Jama*. 319[15], 1566-1579

[28]R. Kones, ***2011***. Primary prevention of coronary heart disease: integration of new data, evolving views, revised goals, and role of rosuvastatin in management. A comprehensive survey[J]. *Drug Des Devel Ther*. 5[325-380

[29]P. M. Ridker, D. L. Bhatt, A. D. Pradhan, R. J. Glynn, J. G. MacFadyen and S. E. Nissen, ***2023***. Inflammation and cholesterol as predictors of cardiovascular events among patients receiving statin therapy: a collaborative analysis of three randomised trials[J]. *Lancet*.

[30]曹佳齐, 郑宏超, 丁跃有, 周志文 and 赵莉芳, ***2020***. 增强型体外反搏治疗对非高危稳定型心绞痛患者疗效及心血管不良事件发生率的影响[J]. *临床和实验医学杂志*. 19[05], 500-502

[31]王晶, 邓松柏, 刘亚杰, 佘强 and 杜建霖, ***2020***. PCSK9 抑制剂对极高危动脉粥样硬化性心血管疾病患者血脂谱的影响[J]. *中华危重病急救医学*. 32[3], 341-344

[32]G. G. Schwartz, P. G. Steg, M. Szarek, D. L. Bhatt, V. A. Bittner, R. Diaz, J. M. Edelberg, S. G. Goodman,

For peer review only - http://bmjopen.bmj.com/site/about/guidelines.xhtml

C. Hanotin, R. A. Harrington, J. W. Jukema, G. Lecorps, K. W. Mahaffey, A. Moryusef, R. Pordy, K. Quintero, M. T. Roe, W. J. Sasiela, J. F. Tamby, P. Tricoci, H. D. White and A. M. Zeiher, ***2018***. Alirocumab and Cardiovascular Outcomes after Acute Coronary Syndrome[J]. *N Engl J Med*. 379[22], 2097-2107

[33]K. Oyama, R. H. M. Furtado, A. Fagundes, Jr., T. A. Zelniker, M. Tang, J. Kuder, S. A. Murphy, A. Hamer, H. Wang, A. C. Keech, R. P. Giugliano, M. S. Sabatine and B. A. Bergmark, ***2021***. Effect of Evolocumab on Complex Coronary Disease Requiring Revascularization[J]. *J Am Coll Cardiol*. 77[3], 259-267

[34]赵娟, 李伟, 郑永强 and 曾肃友, ***2022***. 阿利西尤单抗联合瑞舒伐他汀对急性心肌梗死患者PCI 术后冠脉微循环、炎症及心功能的影响[J]. *临床和实验医学杂志*. 21[24], 2606-2610

[35]R. Ragusa, G. Basta, D. Neglia, R. De Caterina, S. Del Turco and C. Caselli, ***2021***. PCSK9 and atherosclerosis: Looking beyond LDL regulation[J]. *Eur J Clin Invest*. 51[4], e13459

[36]D. Patriki, S. S. S. Saravi, G. G. Camici, L. Liberale and J. H. Beer, ***2022***. PCSK 9: A Link Between Inflammation and Atherosclerosis[J]. *Curr Med Chem*. 29[2], 251-267

[37]H. Tavori, I. Giunzioni, I. M. Predazzi, D. Plubell, A. Shivinsky, J. Miles, R. M. Devay, H. Liang, S. Rashid, M. F. Linton and S. Fazio, ***2016***. Human PCSK9 promotes hepatic lipogenesis and atherosclerosis development via apoE- and LDLR-mediated mechanisms[J]. *Cardiovasc Res*. 110[2], 268-278

[38]Q. Kong, M. Liu, Y. Li, Q. Zhu and G. Su, ***2020***. Effect of evolocumab on the progression and stability of atherosclerotic plaques as evaluated by grayscale and iMAP-IVUS[J]. *Ann Palliat Med*. 9[5], 3078-3088

[39]P. Pęczek, M. Leśniewski, T. Mazurek, L. Szarpak, K. J. Filipiak and A. Gąsecka, ***2021***. Antiplatelet Effects of PCSK9 Inhibitors in Primary Hypercholesterolemia[J]. *Life (Basel)*. 11[6],

[40]M. Ruscica, L. Tokgözoğlu, A. Corsini and C. R. Sirtori, ***2019***. PCSK9 inhibition and inflammation: A narrative review[J]. *Atherosclerosis*. 288[146-155

[41]C. M. Ballantyne, P. Banka, G. Mendez, R. Garcia, J. Rosenstock, A. Rodgers, G. Mendizabal, Y. Mitchel and A. L. Catapano, ***2023***. Efficacy and safety of the oral PCSK9 inhibitor MK-0616: a phase 2b randomized controlled trial[J]. *J Am Coll Cardiol*.

[42]王昱渊, 黎子微, 崔鹏辉 and 武军铎, ***2022***. 新型降脂药物的研究进展[J]. *中国循证心血管医学杂志*. 14[09], 1148-1150

[43]U. Laufs, M. Banach, G. B. J. Mancini, D. Gaudet, L. T. Bloedon, L. R. Sterling, S. Kelly and E. S. G. Stroes, ***2019***. Efficacy and Safety of Bempedoic Acid in Patients With Hypercholesterolemia and Statin Intolerance[J]. *J Am Heart Assoc*. 8[7], e011662

For peer review only - http://bmjopen.bmj.com/site/about/guidelines.xhtml

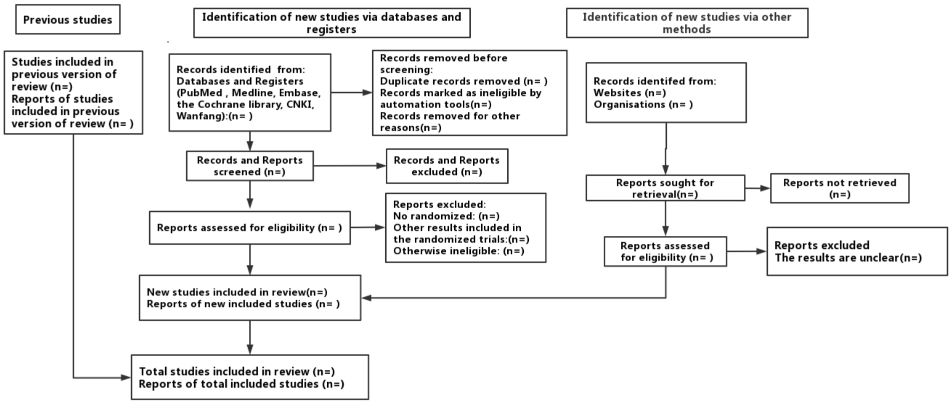

Fig 1 Flow Chart of literature Retrieval

116x52mm (300 x 300 DPI)

General information

Title	Author	Year of publication	RCTs?(Yes/No)

For peer review only

Blind method	Sample size of treatment group	Sample size of control group

For peer review only

Average age Male ratio

For peer review only

Basic information of participant

Proportion of patients with hypertension

For peer review only

ts

Proportion of patients with diabetes	Basic diseases	Follow up time

For peer review only

Basic drug treatment	Type of medication in treatment group

For peer review only

Interventions

Type of medication in control group	Dosage and frequency of medication

For peer review only

Intervention duration	Treatment group:MACEs	Control group:MACEs

For peer review only

Treatment group:LDL-C Control group:LDL-C Treatment group:TC

For peer review only

Control group:TC Treatment group:TG Control group:TG

For peer review only

Outcome indicators

Treatment group:HDL-C Control group:HDL-C

For peer review only

Treatment group:CRP/hs-CRP Control group:CRP/hs-CRP

For peer review only

Treatment group:IL-6 Control group:IL-6 Treatment group:TNF-α

For peer review only

Control group:TNF-α   Treatment group:MMP 9   Control group:MMP 9

For peer review only

Treatment group:LAD Control group:LAD Treatment group:LVEF

For peer review only

Control group:LVEF Treatment group:E/A Control group:E/A

For peer review only

Reporting checklist for protocol of a systematic review and meta analysis.

Based on the PRISMA-P guidelines.

Instructions to authors

Complete this checklist by entering the page numbers from your manuscript where readers will find each of the items listed below.

Your article may not currently address all the items on the checklist. Please modify your text to include the missing information. If you are certain that an item does not apply, please write "n/a" and provide a short explanation.

Upload your completed checklist as an extra file when you submit to a journal.

In your methods section, say that you used the PRISMA-Preporting guidelines, and cite them as:

Moher D, Shamseer L, Clarke M, Ghersi D, Liberati A, Petticrew M, Shekelle P, Stewart LA. Preferred Reporting Items for Systematic Review and Meta-Analysis Protocols (PRISMA-P) 2015 statement. Syst Rev. 2015;4(1):1.

		Reporting Item	Page Number
**Title**			
Identification	#1a	Identify the report as a protocol of a systematic review	1
Update	#1b	If the protocol is for an update of a previous systematic review, identify as such	1
**Registration**			
	#2	If registered, provide the name of the registry (such as PROSPERO) and registration number	2
**Authors**			
Contact	#3a	Provide name, institutional affiliation, e-mail address of all protocol authors; provide physical mailing address of corresponding author	1
Contribution	#3b	Describe contributions of protocol authors and identify the guarantor of the review	20

For peer review only

**Amendments**			
	#4	If the protocol represents an amendment of a previously completed or published protocol, identify as such and list changes; otherwise, state plan for documenting important protocol amendments	n/a,This item is not apply to the protocol.
**Support**			
Sources	#5a	Indicate sources of financial or other support for the review	20
Sponsor	#5b	Provide name for the review funder and / or sponsor	20
Role of sponsor or funder	#5c	Describe roles of funder(s), sponsor(s), and / or institution(s), if any, in developing the protocol	20
**Introduction**			
Rationale	#6	Describe the rationale for the review in the context of what is already known	3-6
Objectives	#7	Provide an explicit statement of the question(s) the review will address with reference to participants, interventions, comparators, and outcomes (PICO)	3-6
**Methods**			
Eligibility criteria	#8	Specify the study characteristics (such as PICO, study design, setting, time frame) and report characteristics (such as years considered, language, publication status) to be used as criteria for eligibility for the review	11-12
Information sources	#9	Describe all intended information sources (such as electronic databases, contact with study authors, trial registers or other grey literature sources) with planned dates of coverage	6-9
Search strategy	#10	Present draft of search strategy to be used for at least one electronic database, including planned limits, such that it could be repeated	8-9
Study records - data management	#11a	Describe the mechanism(s) that will be used to manage records and data throughout the review	7

For peer review only

Study records - selection process	#11b	State the process that will be used for selecting studies (such as two independent reviewers) through each phase of the review (that is, screening, eligibility and inclusion in meta-analysis)	9-11
Study records - data collection process	#11c	Describe planned method of extracting data from reports (such as piloting forms, done independently, in duplicate), any processes for obtaining and confirming data from investigators	11-13
Data items	#12	List and define all variables for which data will be sought (such as PICO items, funding sources), any pre-planned data assumptions and simplifications	11-12
Outcomes and prioritization	#13	List and define all outcomes for which data will be sought, including prioritization of main and additional outcomes, with rationale	12
Risk of bias in individual studies	#14	Describe anticipated methods for assessing risk of bias of individual studies, including whether this will be done at the outcome or study level, or both; state how this information will be used in data synthesis	12-14
Data synthesis	#15a	Describe criteria under which study data will be quantitatively synthesised	12-13
Data synthesis	#15b	If data are appropriate for quantitative synthesis, describe planned summary measures, methods of handling data and methods of combining data from studies, including any planned exploration of consistency (such as I2, Kendall's τ)	12-16
Data synthesis	#15c	Describe any proposed additional analyses (such as sensitivity or subgroup analyses, meta-regression)	15
Data synthesis	#15d	If quantitative synthesis is not appropriate, describe the type of summary planned	12-13
Meta-bias(es)	#16	Specify any planned assessment of meta-bias(es) (such as publication bias across studies, selective reporting within studies)	14
Confidence in cumulative	#17	Describe how the strength of the body of evidence will be assessed (such as GRADE)	15-16

For peer review only

evidence

Notes:

- 4: n/a,This item is not apply to the protocol. The PRISMA-P elaboration and explanation paper is distributed under the terms of the Creative Commons Attribution License CC-BY. This checklist was completed on 26. March 2023 using https://www.goodreports.org/, a tool made by the EQUATOR Network in collaboration with Penelope.ai

For peer review only

BMJ Open

Effects of PCSK9 inhibitors on coronary microcirculation, inflammation and cardiac function in patients with CHD after PCI: A protocol for systematic review and meta-analysis.

Journal:	*BMJ Open*
Manuscript ID	bmjopen-2023-074067.R1
Article Type:	Protocol
Date Submitted by the Author:	26-Jul-2023
Complete List of Authors:	YE, Xuejiao; China Academy of Chinese Medical Sciences, Guang'anmen Hospital<br>Wang, Shihan ; China Academy of Traditional Chinese Medicine Guang'anmen Hospital, Department of Cardiology<br>LIU, Xiao'an; Capital Medical University<br>Wu, Qian; China Academy of Chinese Medical Sciences Guang'anmen Hospital<br>LV, Yanfei; Shanghai Qianhe Technology Co LTD<br>Lv, Qianyu; China Academy of Chinese Medical Sciences Guang'anmen Hospital<br>Li, Junjia; China Academy of Chinese Medical Sciences Guang'anmen Hospital<br>Li, Lanlan; China Academy of Chinese Medical Sciences Guang'anmen Hospital<br>Yang, Yingtian; Beijing University of Chinese Medicine,
**Primary Subject Heading**:	Cardiovascular medicine
Secondary Subject Heading:	Cardiovascular medicine
Keywords:	Coronary heart disease < CARDIOLOGY, Pharmacology < TROPICAL MEDICINE, Cardiac surgery < SURGERY, Coronary intervention < CARDIOLOGY, Ischaemic heart disease < CARDIOLOGY, Myocardial infarction < CARDIOLOGY

SCHOLARONE™
Manuscripts

Effects of PCSK9 inhibitors on coronary microcirculation, inflammation and cardiac function in patients with CHD after PCI: A protocol for systematic review and meta-analysis.

Xuejiao YE[1], Shihan WANG[1*], Xiao'an LIU[2], Qian WU[1], Yanfei LV[3], Qianyu LV[1], Junjia LI[1], Lanlan LI[1], Yingtian YANG[1]

1 Guang'anmen Hospital, Chinese Academy of Chinese Medical Sciences, Beijing, China, 2 Capital University of Medical, Beijing, China, 3 Shanghai Qianhe Technology Co., LTD

*CORRESPONDENCE: Shihan Wang.
Email address: wangshihan91@126.com.
Affiliations: Guang'anmen Hospital, Chinese Academy of Chinese Medical Sciences, Beijing, China.

Abstract:

Introduction: Coronary heart disease(CHD) is one of the common cardiovascular diseases that seriously jeopardize human health, and endothelial inflammation and dyslipidemia are the initiating links leading to its occurrence. Percutaneous coronary intervention(PCI) is one of the most effective surgical treatments for CHD with narrowed or blocked blood vessels, which can quickly unblock the blocked vessels and restore coronary blood supply. However, most patients may experience coronary microcirculation disorders(CMD) and decreased cardiac function after PCI treatment, which directly affects the efficacy of PCI and the prognosis of patients. Preprotein converting enzyme subtilisin/Kexin 9(PCSK9) inhibitors

For peer review only - http://bmjopen.bmj.com/site/about/guidelines.xhtml

are novel pleiotropy lipid-lowering drug with dual anti-inflammation and lipid-lowering effects, and represent a new clinical pathway for rapid correction of dyslipidemia. Therefore, we designed this protocol to systematically evaluate the effects of PCSK9 inhibitors on coronary microcirculation and cardiac function in patients with CHD after PCI, and to provide high-quality evidence-based evidence for the clinical application of PCSK9 inhibitors.

Methods and analysis: This protocol is reported strictly in accordance with the 2020 Preferred Reporting Items for Systematic Reviews and Meta-analyses Protocols Guidelines (PRISMA-P). This protocol had been registered in PROSPERO (Registration number CRD42022346189). We will search PubMed, EMBASE, Web of Science, and three Chinese databases(CNKI, Wanfang, and VIP database) according to preset search strategies, without language and publication data restrictions. We will work with manual retrieval to screen references that have been included in the literature. Google Scholar will be used to search for grey literature. The final included literature must meet the established inclusion criteria. Titles, abstracts and full text will be extracted independently by two reviewers, and disagreements will be resolved through discussion or the involvement of a third reviewer. Extracted data will be analyzed using Review Manager 5.3. The Cochrane Risk of Bias Tool will be used to evaluate

For peer review only - http://bmjopen.bmj.com/site/about/guidelines.xhtml

the risk of bias. Publication bias will be assessed by funnel plots. Heterogeneity will be assessed by I^2 test and subgroup analyses will be used to further investigate potential sources of heterogeneity. The quality of the literature will be assessed by GRADE score. This protocol will start in January 2026 and end in December 2030.

PROSPERO registration number: CRD42022346189

Ethics and dissemination: This study is a systematic review of published literature data and no special ethical approval was required.

Keywords: PCSK9 inhibitors, PCI, coronary microcirculation, inflammation, cardiac function.

Strengths and limitations of this study:

- To the best of our knowledge, this protocol is an updated systematic review and meta-analysis designed to assess the effects of PCSK9 inhibitors on coronary microcirculation, inflammation and cardiac function in patients with CHD after PCI.
- Compared with the previous studies, we considered a wider range of outcome indicators, including laboratory indicators and ultrasound indicators.
- Subgroup and sensitivity analyses will be used to explore potential heterogeneity.
- The limited number of studies that meet our inclusion criteria is a limitation of this study.

For peer review only - http://bmjopen.bmj.com/site/about/guidelines.xhtml

1. Introduction

Coronary heart disease(CHD) is a cardiovascular disease caused by coronary atherosclerosis, which causes narrowing or occlusion of the lumen of the blood vessels, resulting in myocardial ischemia, hypoxia, or necrosis(1). Percutaneous coronary intervention (PCI), coronary artery bypass grafting(CABG), and lipid management are three effective treatments for CHD. And PCI, in particular, is the mainstay of treatment for coronary artery multibranch vasculopathy in the clinic. It is widely used in clinic practice(2). It can effectively unblock the blocked vessels and realize the reconstruction of coronary blood flow(3). Accompanying with obvious benefits, there are also certain risks. Invasive procedures such as balloon dilation during PCI can damage vascular endothelium, induce an inflammation in endothelial cells, and promote micro-thrombosis, leading to coronary microcirculatory disorders(CMD) and myocardial ischemia-reperfusion injury(MIRI), which significantly increase the risk of major adverse cardiovascular events(MACEs) in patients during the perioperative period(4, 5).

The coronary microcirculation consists of micro-vessels with diameters of <500μm(microarterioles, capillaries and microvessels), which collectively participate in maintaining the normal physiological function of cardiomyocytes and the stability of the coronary

For peer review only - http://bmjopen.bmj.com/site/about/guidelines.xhtml

microcirculation. CMD refers to the structural and functional abnormalities of the coronary vasculature, and endovascular obstruction and extravascular compression are the basis for the occurrence of CMD(6). When coronary angiography is normal and there are no other diseases, CMD is usually the main reason for the occurrence of myocardial ischemia(7). The ISCHEMIA study published at the 2019 American Heart Association meeting showed that the risk of MACEs in CHD patients with moderate myocardial ischemia is not significantly reduced after completion of revascularization, as expected(8). Moreover, in conjunction with clinical practice, we found that some patients with CHD did not experience improvement in angina symptoms after PCI, nor did the myocardium recover to full reperfusion levels. All of these studies suggest that potential CMD may be responsible for influencing the prognosis of patients with CHD(9). Due to the fact that coronary angiography can only detect 5% of coronary branches, and the remaining 95% of coronary microcirculation cannot be clearly visualized, CMD is easily misdiagnosed and greatly increases the risk of MACEs(10). Therefore, clarifying the underlying mechanisms of microvascular injury after PCI is of great clinical significance in identifying therapeutic targets for CMD.

TIMI classification is one of the indicators to evaluate the recovery of

For peer review only - http://bmjopen.bmj.com/site/about/guidelines.xhtml

coronary artery flow reperfusion after PCI. It has been found that the increase in serum hs-CRP concentration after PCI is negatively correlated with the coronary TIMI flow classification. This suggest that inflammation may lead to a decrease in TIMI classification and poor recovery of coronary blood flow(11). In addition, the extracellular matrix(ECM) is important for maintaining the structural stability of cardiomyocytes. Matrix metalloproteinases(MMPs) and tissue inhibitor of metalloproteinase(TIMP) play a key role in the maintenance of ECM stability (12, 13). Matrix metalloproteinases -9(MMP-9) is an inflammation marker that breaks down collagen in the plaque fibrous cap, causing the fibrous cap of atheromatous plaques to become thinner and easier to rupture, which in turn destabilizes the plaque and induces thrombosis(14). Studies has confirmed that the activity of MMP-9 in unstable plaques is 3-5 times higher than that in stable plaques. This suggests that the higher the activity of MMP-9, the stronger the degradation of ECM and the easier the plaques rupture(15). TIMP is an inhibitor of MMPs, which is expressed by smooth muscle cells under physiological conditions. TIMP plays an important role in preventing ventricular remodeling after myocardial ischemia by inhibiting the biological activity of MMPs and reducing the degradation of the ECM. The balance between MMP-9 and TIMP jointly maintains the stability of

For peer review only - http://bmjopen.bmj.com/site/about/guidelines.xhtml

atherosclerotic plaques. When MMP-9 levels decrease and TIMP levels increase, ECM degradation decreases and plaques become more stable. Conversely, ECM degradation increases, and plaques become destabilized and more prone to rupture, increasing the risk of acute coronary events(16). Based on this, we hypothesized that inflammation factors such as MMP-9 may be biomarkers for assessing the prognosis of patients with CHD and may become new therapeutic targets.

Currently, several clinical studies have evaluated the cardioprotective effects of PCSK9 inhibitors after PCI, but there is no relevant systematic literature review on this topic. Therefore, we designed this protocol to systematically summarize the effects of PCSK9 inhibitors on coronary microcirculation and cardiac function in patients with CHD after PCI, and to provide high-quality evidence-based medical evidence for the clinical application of PCSK9 inhibitors.

2. Objective

① Main efficacy indicators: TIMI flow classification of coronary arteries, myocardial injury markers(cardiac troponin(cTn), creatine kinase isoenzyme(CK-MB), myoglobin(Myo)), N-terminal pro-Brain Natriuretic Peptide levels(NT-pro BNP) and D-dimer(D-D) before and after PCI.

For peer review only - http://bmjopen.bmj.com/site/about/guidelines.xhtml

② Secondary efficacy indicators: Serum inflammation markers (CRP/high-sensitivity CRP(hs-CRP), interleukin(IL-6), tumor necrosis factor-α (TNF-α), matrix metalloproteinase-9(MMP-9))，cardiac function indicators(left ventricular ejection fraction(LVEF), fractional shortening(FS)), blood lipid indicators(LDL-C, total cholesterol(TC), triglyceride(TG), high-density lipoprotein cholesterol(HDL-C)).

③ Safety indicators: The incidence of postoperative complications (puncture point hematoma, gingival bleeding, hematochezia, urinary blood, etc), myocardial ischemia reperfusion injury(MIRI)(reperfusion arrhythmia(RA), myocardial stunning, no-reflow and lethal reperfusion injury, etc), MACEs(incidence of recurrent myocardial infarction, heart failure, cardiogenic shock, and cardiogenic death within six months after PCI(17, 18))(19).

3. Methods and analysis

3.1 Protocol registration

This protocol is reported following the 2020 Preferred Reporting Items for Systematic Review and Meta-analysis Protocols statement(PRISMA-P)(20) and has been registered on the International Prospective Register of Systematic Reviews(PROSPERO) network. Registration number is

For peer review only

For peer review only - http://bmjopen.bmj.com/site/about/guidelines.xhtml

CRD4202346189.

3.2 Date sources and search strategies

For this article, we will search PubMed, EMBASE, Web of Science, and three Chinese databases(CNKI, Wanfang, VIP database). Google Scholar will be used to search for grey literature. We will work with manual retrieval to screen references that have been included in the literature.

Search terms are combinations of free and subject terms, including PCI, PCSK9 inhibitors, inflammation, CHD, etc. Search terms are connected by Boolean operators. We will provide a detailed search strategy for PubMed as shown in Fig 1. For other databases, these terms will be modified as need, and detailed search strategy as shown in supplementary file supplementary file.pdf. There are no language or data restrictions. Duplicates will be automatically removed by EndNote (version X9, Clarivate Analytics), and the remaining duplicates will be removed manually by comparing authors, titles and publication dates. All literature that meets the inclusion criteria will be further screened by reading the full text to extract the required data, and excluded literature will be required to document the reasons. This process will be conducted independently by two reviewers and any issues and disagreements will be resolved through mutual discussion or discussion with a third reviewer. In accordance with the PRISMA-P

For peer review only - http://bmjopen.bmj.com/site/about/guidelines.xhtml

guidelines, we will summarize the entire study screening process in a flowchart.

Fig 1. Search strategy for PubMed

PCSK9 inhibitors [MeSH]

1. (proprotein convertase subtilisin/kexin type 9 monoclonal antibody) OR (PCSK9 inhibitors) OR (evolocumab) OR (alirocumab) OR (Praluent) OR (Repatha) [Title/Abstract]

Inflammation [MeSH]

2. (Inflammation) OR (Inflammatory response) OR (Inflammation reaction) [Title/Abstract]

Randomized controlled trials [MeSH]

3. (RCT) OR (RCTs) OR (Randomized controlled trials) OR (Randomized controlled trial) [Title/Abstract]

Coronary heart disease [MeSH]

4. (Coronary artery disease) OR (Arteriosclerotic heart disease) OR (CAD) OR (CHD) OR (acute coronary syndrome) OR (chronic coronary syndrome) OR (ACS) OR (CCS) [Title/Abstract]

5. 1 and 2 and 3 and 4

3.3 Eligibility criteria for study selection

3.3.1 Study design

RCTs comparing the effects of PCSK9 inhibitors on coronary microcirculation, inflammation factors, and cardiac function in

For peer review only

For peer review only - http://bmjopen.bmj.com/site/about/guidelines.xhtml

patients with CHD after PCI will be included without time and language restrictions. Non-randomized controlled trials, observational studies, animal experiments, case report, reviews and conference abstracts will be excluded. This protocol will begin in January 2026 and end in December 2030.

3.3.2 Participants

Inclusion criteria:

① Male or female patients who meet the diagnostic criteria for CHD(Definition criteria of CHD: Acute coronary syndrome(ACS) and Chronic coronary syndrome(CCS))(21), aged18-65(22-24).

② PCI completed within 72 hours.

③ Patients who are able to tolerant and willing to receive PCSK9 inhibitors or statins or ezetimibe lipid-lowering treatment.

Exclusion criteria:

① Intolerant to statins, ezetimibe or PCSK9 inhibitors.

② Severe liver and kidney dysfunction.

③ Pregnant or lactating women.

④ Malignant tumor or blood system disease.

⑤ History of severe allergic reactions to lipid-lowering drugs.

⑥ Unsuitable participants for any other reasons, as judged by the reviewers.

3.3.3 Types of interventions

For peer review only - http://bmjopen.bmj.com/site/about/guidelines.xhtml

Any RCTs using PCSK9 inhibitors before PCI will be included. PCSK9 inhibitors include evolocumab or alirocumab at a dose of 140mg/2weeks or 420mg/4weeks. Interventions included but not limited to the following:

① PCSK9 inhibitors VS Placebo;

② PCSK9 inhibitors VS Statins;

③ PCSK9 inhibitors＋Statins VS Statins;

④ PCSK9 inhibitors VS Statins ＋ Ezetimibe;

⑤ PCSK9 inhibitors ＋ Statins VS Statins ＋ Ezetimibe;

⑥ PCSK9 inhibitors ＋ Statins VS Ezetimibe＋Statins.

3.3.4 Types of outcome measures Eligible studies should report at least one of the following outcomes.

① Main efficacy indicators: TIMI flow classification of coronary arteries, myocardial injury markers(cTn, CK-MB, Myo), NT-pro BNP and D-D before and after PCI.

② Secondary efficacy indicators: Serum inflammation markers (CRP/hs-CRP, IL-6, TNF-α, MMP-9), cardiac function indicators (LVEF, FS), blood lipid indicators(LDL-C, TC, TG, HDL-C).

③ Safety indicators: The incidence of postoperative complications (puncture point hematoma, gingival bleeding, hematochezia, urinary blood, etc), MIRI (RA, myocardial stunning, no-reflow and lethal reperfusion injury, etc), MACEs(incidence of recurrent

For peer review only

myocardial infarction, heart failure, cardiogenic shock, and cardiogenic death within six months after PCI(17, 18))(19).

3.4 Data collection and analysis

3.4.1 Date extraction and management

After screening the literature according to the inclusion and exclusion criteria, we will perform data extraction using a self-made Microsoft Excel data extraction form, which presented in supplementary material. This process will be performed independently by two reviewers, and any disagreements, if any, will be resolved through mutual discussion or discussion with a third reviewer. The extracted data are presented below:

① Study identification: Title, first author's name, year of publication.

② Study characteristics: Study design (RCTs), duration of follow-up, number of cases.

③ Study population: Sample size, mean or median age, gender composition ratio, proportion of patients with diabetes and hypertension, basic disease, basic medication.

④ Interventions: Type, dose and frequency of medication, course of treatment.

⑤ Main efficacy indicators: TIMI flow classification of coronary arteries, myocardial injury markers(c Tn, CK-MB, Myo), NT-pro BNP and D-D before and after PCI.

For peer review only - http://bmjopen.bmj.com/site/about/guidelines.xhtml

⑥ Secondary efficacy indicators: Serum inflammation indicators(CRP/hs-CRP, IL-6, TNF-α, MMP-9), cardiac function indicators(LVEF, FS), blood lipid indicators(LDL-C, TC, TG, HDL-C).

⑦ Safety indicators: The incidence of postoperative complications (puncture point hematoma, gingival bleeding, hematochezia, urinary blood, etc), MIRI(RA, myocardial stunning, no-reflow and lethal reperfusion injury, etc), MACEs(incidence of recurrent myocardial infarction, heart failure, cardiogenic shock, and cardiogenic death within six months after PCI(17, 18))(19).

3.4.2 Data synthesis and analysis

We will apply Review Manager 5.3 provided by the Cochrane Collaboration(http://www-cochrane-org.vpn1.hactcm.edu.cn/) to analyze the obtained data(25). For continuous variables, mean value (MD) and 95% confidence intervals(CI) will be used for data analysis. For dichotomous variables, odds ratio(OR) and 95%CI will be applied. $P \geq 0.05$ indicates no statistical significance, and $P<0.05$ indicates statistical significance. The heterogeneity of the literature will be evaluated by the I^2 test. If $P \geq 0.1$ and $I^2 \leq 50\%$, there is no statistical heterogeneity, and the fixed effect model will be used. If $P < 0.1$ and $I^2 > 50\%$, indicates statistical heterogeneity exists, and random effect model will be used.

For peer review only - http://bmjopen.bmj.com/site/about/guidelines.xhtml

3.5 Dealing with missing data

For incomplete or missing data, we will contact the corresponding author to obtain it. Otherwise, relevant literature will be excluded.

3.6 Risk assessment of included literature

The Cochrane Collaboration's risk of bias assessment tool(http://www-cochrane-org.vpn1.hactcm.edu.cn/) will be applied to independently assess the bias risk of each included literature(26). We will assess the risk of bias from six domains: performance bias (blinding of participants and personnel), selection bias (random sequence generation, allocation concealment), detection bias (blinding of outcome assessment), attrition bias (incomplete outcome data), reporting bias (selective reporting) and other potential biases. The quality of assessment is divided into three grades: low risk of bias, some concerns, high risk of bias. For duplicate literature, we will select only the original version. Any doubts and disagreements will be discussed and resolved by two reviewers, and if necessary, we will seek the help of a third reviewer. Review Manager 5.3 will be used to draw charts for risk assessment.

3.7 Publication of bias

If more than 10 literature were included, We will use the funnel plot to visually assess publication bias. The symmetry of the two sides of the funnel plot indicates the absence of publication bias, while

For peer review only - http://bmjopen.bmj.com/site/about/guidelines.xhtml

asymmetric of the distribution of the two sides indicates the possible existence of publication bias. If the number of literature was less than 10, Egger's test will be used for statistical analysis. $P<0.05$ indicates the existence of publication bias, and $P\geqslant0.05$ indicates the absence of publication bias.

3.8 Data analysis

3.8.1 Assessment of heterogeneity

We will use the I^2 test to evaluate the heterogeneity of the included literature, with $I^2\leqslant 50\%$ indicating low heterogeneity and $I^2>50\%$ indicating high heterogeneity.

3.8.2 Subgroup analysis and the sources of heterogeneity

When heterogeneity is evident, we will conduct subgroup analysis to investigate the source of heterogeneity:

① Age.

② Race.

③ Proportion of patients with hypertension or diabetes.

④ Types and doses of interventions.

3.8.3 Sensitivity analyses

We will use a one by one exclusion method for sensitivity analysis to evaluate the robustness of the study results. Sensitivity analysis needs to consider various uncertainties, such as small sample size, missing data, and methodological quality. After excluding low-quality

For peer review only - http://bmjopen.bmj.com/site/about/guidelines.xhtml

literature, we need to repeat the analysis.

3.8.4 Quality evaluation of included literature

We will use GRADE score(27) to evaluate the quality of included literature, and each literature will be stratified according to high-level of evidence, moderate level of evidence, low-level of evidence and very low-level evidence.

3.9 Patient and public involvement

Since this study is a secondary study based on other studies, there will be no direct patient or public involvement in this study.

4. Discussion

PCI can effectively unblock the blocked vessels, rapidly restore coronary blood flow and improve myocardial blood supply. However, invasive procedures such as stenting, balloon dilation, and myocardial ischemia reperfusion during PCI can damage the endothelium by inducing platelet aggregation, microvascular spasm, inflammation, and myocardial interstitial edema, among other mechanisms(6, 28). MIRI leads to the overproduction of reactive oxygen species(ROS), which induces oxidative stress, inflammation, and endothelial dysfunction, which are the key pathogenic mechanisms that lead to the development of CMD(29). Bruins et al.(30) proved the existence of inflammation by measuring indicators such as CRP and IL-6 in the

For peer review only - http://bmjopen.bmj.com/site/about/guidelines.xhtml

early postoperative period. BAKTASHIANM et al.(31) found that an increase in the serum inflammation factor, hs-CRP, was strongly related with the occurrence of in-stent restenosis. Yang et al.(32) found that hs-CRP level was positively correlated with the coefficient of resistance(IMR) of coronary microcirculation after PCI. Recent studies have further confirmed that elevated levels of inflammation factor CRP can directly damage the diastolic function of the vascular endothelium and stimulate the secretion of MMPs and other plaque rupture-promoting substances, thereby interfering with coronary perfusion(33). All of these studies suggest a potential link between the inflammation and CMD after PCI. Inflammation induces endothelial cell activation and increases ROS production, indirectly promoting the activation and adhesion of platelet(34). In addition, inflammation and myocardial ischemia reperfusion can cause vascular endothelial cell injury and increase the permeability of the microvascular walls, which in turn leads to edema of myocardial tissues, compression of coronary micro-vessels, and increased microcirculation resistance(35). Endothelial cell injury also activates the body's coagulation mechanism, induces platelet adhesion and aggregation at the site of endothelial injury, and promotes thrombus formation, further aggravating CMD and increasing the risk of in-stent restenosis events(36).

For peer review only - http://bmjopen.bmj.com/site/about/guidelines.xhtml

Dyslipidemia is not only an important independent risk factor for CHD, but also an important initiator of coronary atheromatous plaque inflammation. It can affect coronary microcirculation through various mechanisms(37).Large lipid accumulations in the vascular lumen can induce inflammation, endothelial cell injury, ECM remodeling, platelet activation, and thrombosis, which can directly lead to coronary artery stenosis or occlusion(38). In addition, higher lipid levels can stimulate the vascular endothelium and induce apoptosis of endothelial cell, which ultimately results in coronary atheromatous plaque development. A prospective cohort study(39) found that patients with CMD had high serum TC and LDL-C levels, indicating that hyperlipidemia may contribute to microvascular injury and CMD. Serum LDL-C level was positively associated with atherosclerosis plaque formation and the occurrence of MACEs(40). For every 1mmol/L reduction in LDL-C, there is a 22% reduction in MACEs, a 10% reduction in all-cause mortality, and a 20% reduction in the risk of CHD mortality (41). Effective control of lipids, therefore, is critical to attenuating inflammation and preventing the development of MACEs after PCI(42). Intensive lipid-lowering therapy, especially lowering serum LDL-C, is important for improving the prognosis of patients with CHD(43).

Anti-inflammation and lipid management are important interventions

For peer review only - http://bmjopen.bmj.com/site/about/guidelines.xhtml

for patients with CHD after PCI, and are key to ensuring smooth coronary microcirculation and preventing in-stent thrombosis. Statins are the cornerstone of the treatment of CHD. Their dual lipid-lowering and anti-inflammation effects are key to their positive effect on coronary atherosclerosis(44). However, due to the existence of “the 6% effect” (45), doubling the dose of statins can only reduce LDL-C level by a further 6%. adverse effects, such as rhabdomyolysis and hepatic dysfunction, limit the clinical application of statins(46). As a secreted serine protease, PCSK9 is mainly synthesized and secreted by the liver. It can promote plaque inflammation by interfering with circulating lipid levels or increasing monocyte infiltration and differentiation(47-49). PCSK9 inhibitors are a new type of non-statin lipid-lowering drugs. On the one hand, it exerts lipid-lowering and plaque stabilizing effects by decreasing the degradation of LDL-R to increase the clearance of LDL-R. On the other hand, it exerts anti-inflammation effects by reducing LDL-C levels and LDL-R mediated platelet activation(50, 51). Both can reduce the risk of MACEs in patients with CHD, and both are safe and well tolerated by the patients(52-54). In addition, PCSK9 inhibitors have a synergistic effect with statins. ODYSSEY-LONG TERM trial showed that the combination of PCSK9 inhibitors and statins could reduce the risk of MACEs by about 50% in patients with high-risk cardiovascular

For peer review only - http://bmjopen.bmj.com/site/about/guidelines.xhtml

diseases, which provides a theoretical basis for the safety of clinical application of PCSK9 inhibitors(55, 56). Based on this, we believe that the powerful lipid-lowering and potential anti-inflammation effects of PCSK9 inhibitors could promote the improvement of coronary microcirculation and cardiac function after PCI. This conclusion remains to be confirmed by numerous animal and clinical studies(57).

5. Ethics and dissemination

This study is a systematic review of published literature data and no special ethical approval was required.

6. Author contributions

XY and SW designed the study. QW, QL, JL created the tables and figures. XY and YL wrote the initial draft of the manuscript. YY, XL and LL revised the manuscript. All authors contributed to the article and approved the final version of the manuscript.

7. Funding

This research is supported by Beijing Municipal Science & Technology Commission (Z191100006619025).

For peer review only - http://bmjopen.bmj.com/site/about/guidelines.xhtml

8. Conflict of interest

The authors declare that the research was conducted in the absence of any commercial or financial relationships that could be construed as a potential conflict of interest.

9. Supplementary material

The Supplementary Material for this self-made Microsoft Excel data extraction table can be found online at: Microsoft Excel 数据提取表.xlsx

References:

1. Malakar AK, Choudhury D, Halder B, Paul P, Uddin A, Chakraborty S. A review on coronary artery disease, its risk factors, and therapeutics. J Cell Physiol. 2019;234(10):16812-23. Epub 20190220. doi: 10.1002/jcp.28350. PubMed PMID: 30790284.

2. Sá MP, Ferraz PE, Escobar RR, Nunes EO, Soares AM, Araújo e Sá FB, et al. Five-year outcomes following PCI with DES versus CABG for unprotected LM coronary lesions: meta-analysis and meta-regression of 2914 patients. Rev Bras Cir Cardiovasc. 2013;28(1):83-92. doi: 10.5935/1678-9741.20130013. PubMed PMID: 23739937.

3. Gibson CM, Mehran R, Bode C, Halperin J, Verheugt FW, Wildgoose P, et al. Prevention of Bleeding in Patients with Atrial Fibrillation Undergoing PCI. N Engl J Med. 2016;375(25):2423-34. Epub 20161114. doi: 10.1056/NEJMoa1611594. PubMed PMID: 27959713.

4. 林珑，刘冠男，高丽霓，王吟春，李晓东，孔晓琳, et al. 经皮冠状动脉介入术后主要不良心脏事件危险因素研究进展. 临床急诊杂志. 2020;21(11):918-22. doi: 10.13201/j.issn.1009-5918.2020.11.014.

5. Choi KH, Lee JM, Kim SR, Kim D, Choi JO, Kim SJ, et al. Prognostic Value of the Index of Microcirculatory Resistance Over Serum Biomarkers in Cardiac Amyloidosis. J Am Coll Cardiol. 2020;75(5):560-1. doi: 10.1016/j.jacc.2019.11.045. PubMed PMID: 32029139.

6. Sezer M, van Royen N, Umman B, Bugra Z, Bulluck H, Hausenloy DJ, et al. Coronary Microvascular Injury in Reperfused Acute Myocardial Infarction: A View From an Integrative Perspective. J Am Heart Assoc. 2018;7(21):e009949. doi: 10.1161/jaha.118.009949. PubMed PMID: 30608201; PubMed Central PMCID: PMC6404180.

7. 凌浩，刘明明，于雪. 冠状动脉微血管功能障碍研究进展. 国际心血管病杂志. 2022;49(02):65-8.

For peer review only - http://bmjopen.bmj.com/site/about/guidelines.xhtml

8. Padro T, Manfrini O, Bugiardini R, Canty J, Cenko E, De Luca G, et al. ESC Working Group on Coronary Pathophysiology and Microcirculation position paper on 'coronary microvascular dysfunction in cardiovascular disease'. Cardiovasc Res. 2020;116(4):741-55. doi: 10.1093/cvr/cvaa003. PubMed PMID: 32034397; PubMed Central PMCID: PMC7825482.

9. 王澳龙，卫靖靖，于瑞，彭广操，李兴渊，朱明军．经皮冠状动脉介入术术后冠脉微循环障碍的中医药治疗研究概述．中华中医药学刊.1-9.

10. 李威亚，马欢，耿庆山．冠状动脉微循环障碍危险因素及机制研究进展．心血管病学进展. 2021;42(12):1064-8+80. doi: 10.16806/j.cnki.issn.1004-3934.2021.12.003.

11. 陈万林，李垚，杨平，白志生，郑广生．急性心肌梗死患者不同心肌梗死溶栓试验血流分级与炎症指标及光学相干断层扫描所见斑块性质之间的关系．岭南心血管病杂志. 2023;29(01):6-10+34.

12. DeLeon-Pennell KY, Meschiari CA, Jung M, Lindsey ML. Matrix Metalloproteinases in Myocardial Infarction and Heart Failure. Prog Mol Biol Transl Sci. 2017;147:75-100. Epub 20170318. doi: 10.1016/bs.pmbts.2017.02.001. PubMed PMID: 28413032; PubMed Central PMCID: PMC5576003.

13. 刘金龙，张永和．中药对冠心病的治疗作用及其机制探究．长春中医药大学学报. 2019;35(03):589-92. doi: 10.13463/j.cnki.cczyy.2019.03.052.

14. 李七一，韩旭，夏卫军，赖仁胜，陈晓虎，郭宏敏, et al. 通心络胶囊干预载脂蛋白 E 基因敲除小鼠 MMP-1、MMP-9 及 TIMP1 的研究．南京中医药大学学报. 2010;26(06):431-3. doi: 10.14148/j.issn.1672-0482.2010.06.015.

15. 黄婕，杜成林，韦方练，陈诗佳，黄骥．血清 MMP-13、TIMP-1 表达与慢性心力衰竭心室重构的关系．河北医药. 2019;41(08):1186-9.

16. 哈略，崔莹雪，林瑶，左滢竹，赵百孝．艾灸及艾烟对 APOE~(-/-)小鼠动脉粥样硬化斑块稳定性的影响．中华中医药杂志. 2019;34(07):3197-200.

17. De Luca L, Rubboli A, Bolognese L, Uguccioni M, Lucci D, Blengino S, et al. Is percutaneous coronary intervention safe during uninterrupted direct oral anticoagulant therapy in patients with atrial fibrillation and acute coronary syndromes? Open Heart. 2021;8(2). doi: 10.1136/openhrt-2021-001677. PubMed PMID: 34261777; PubMed Central PMCID: PMC8281094.

18. Villanueva DLE, Tiongson MD, Ramos JD, Llanes EJ. Monocyte to High-Density Lipoprotein Ratio (MHR) as a predictor of mortality and Major Adverse Cardiovascular Events (MACE) among ST Elevation Myocardial Infarction (STEMI) patients undergoing primary percutaneous coronary intervention: a meta-analysis. Lipids Health Dis. 2020;19(1):55. Epub 20200326. doi: 10.1186/s12944-020-01242-6. PubMed PMID: 32216795; PubMed Central PMCID: PMC7098114.

19. Alex Chau YL, Won Yoo J, Chuen Yuen H, Bin Waleed K, Chang D, Liu T, et al. The impact of post-operative atrial fibrillation on outcomes in coronary artery bypass graft and combined procedures. J Geriatr Cardiol. 2021;18(5):319-26. doi: 10.11909/j.issn.1671-5411.2021.05.005. PubMed PMID: 34149821; PubMed Central PMCID: PMC8185439.

20. Page MJ, McKenzie JE, Bossuyt PM, Boutron I, Hoffmann TC, Mulrow CD, et al. The PRISMA 2020 statement: An updated guideline for reporting systematic reviews. PLoS Med. 2021;18(3):e1003583. Epub 20210329. doi: 10.1371/journal.pmed.1003583. PubMed PMID: 33780438; PubMed Central PMCID: PMC8007028.

21. Corrigendum to: 2019 ESC Guidelines on diabetes, pre-diabetes, and cardiovascular diseases developed in collaboration with the EASD. Eur Heart J. 2020;41(45):4317. doi: 10.1093/eurheartj/ehz828. PubMed PMID: 31837138.

22. Ferrari R, Abergel H, Ford I, Fox KM, Greenlaw N, Steg PG, et al. Gender- and age-related

For peer review only - http://bmjopen.bmj.com/site/about/guidelines.xhtml

differences in clinical presentation and management of outpatients with stable coronary artery disease. Int J Cardiol. 2013;167(6):2938-43. Epub 20120915. doi: 10.1016/j.ijcard.2012.08.013. PubMed PMID: 22985742.
23. 周学中, 王惠, 罗太阳. 经皮冠脉介入治疗老年冠心病患者的预后及其影响因素. 江苏大学学报(医学版). 2018;28(06):512-5. doi: 10.13312/j.issn.1671-7783.y180224.
24. 葛均波, 张峰. 老年冠心病介入治疗的特殊问题:适应证、耐受性、安全性和有效性. 老年医学与保健. 2007(05):263-5.
25. Cumpston M, Li T, Page MJ, Chandler J, Welch VA, Higgins JP, et al. Updated guidance for trusted systematic reviews: a new edition of the Cochrane Handbook for Systematic Reviews of Interventions. Cochrane Database Syst Rev. 2019;10:Ed000142. doi: 10.1002/14651858.Ed000142. PubMed PMID: 31643080.
26. Sterne JAC, Savović J, Page MJ, Elbers RG, Blencowe NS, Boutron I, et al. RoB 2: a revised tool for assessing risk of bias in randomised trials. Bmj. 2019;366:l4898. Epub 20190828. doi: 10.1136/bmj.l4898. PubMed PMID: 31462531.
27. Guyatt GH, Oxman AD, Vist GE, Kunz R, Falck-Ytter Y, Alonso-Coello P, et al. GRADE: an emerging consensus on rating quality of evidence and strength of recommendations. Bmj. 2008;336(7650):924-6. doi: 10.1136/bmj.39489.470347.AD. PubMed PMID: 18436948; PubMed Central PMCID: PMC2335261.
28. Fröhlich GM, Meier P, White SK, Yellon DM, Hausenloy DJ. Myocardial reperfusion injury: looking beyond primary PCI. Eur Heart J. 2013;34(23):1714-22. Epub 20130327. doi: 10.1093/eurheartj/eht090. PubMed PMID: 23536610.
29. Masi S, Rizzoni D, Taddei S, Widmer RJ, Montezano AC, Lüscher TF, et al. Assessment and pathophysiology of microvascular disease: recent progress and clinical implications. Eur Heart J. 2021;42(26):2590-604. doi: 10.1093/eurheartj/ehaa857. PubMed PMID: 33257973; PubMed Central PMCID: PMC8266605.
30. Bruins P, te Velthuis H, Yazdanbakhsh AP, Jansen PG, van Hardevelt FW, de Beaumont EM, et al. Activation of the complement system during and after cardiopulmonary bypass surgery: postsurgery activation involves C-reactive protein and is associated with postoperative arrhythmia. Circulation. 1997;96(10):3542-8. doi: 10.1161/01.cir.96.10.3542. PubMed PMID: 9396453.
31. Baktashian M, Saffar Soflaei S, Kosari N, Salehi M, Khosravi A, Ahmadinejad M, et al. Association of high level of hs-CRP with in-stent restenosis: A case-control study. Cardiovasc Revasc Med. 2019;20(7):583-7. Epub 20180818. doi: 10.1016/j.carrev.2018.08.015. PubMed PMID: 30232022.
32. 杨宇, 梁梅冰, 黄石安, 许顶立. 经皮腔内冠状动脉介入术对冠状动脉微循环阻力指数及高敏 C 反应蛋白的影响. 中国现代医学杂志. 2014;24(23):59-62.
33. Oikonomou E, Leopoulou M, Theofilis P, Antonopoulos AS, Siasos G, Latsios G, et al. A link between inflammation and thrombosis in atherosclerotic cardiovascular diseases: Clinical and therapeutic implications. Atherosclerosis. 2020;309:16-26. Epub 20200802. doi: 10.1016/j.atherosclerosis.2020.07.027. PubMed PMID: 32858395.
34. Mundi S, Massaro M, Scoditti E, Carluccio MA, van Hinsbergh VWM, Iruela-Arispe ML, et al. Endothelial permeability, LDL deposition, and cardiovascular risk factors-a review. Cardiovasc Res. 2018;114(1):35-52. doi: 10.1093/cvr/cvx226. PubMed PMID: 29228169; PubMed Central PMCID: PMC7729208.
35. 刘磊, 姚道阔, 陈晖. 冠状动脉微循环障碍发病机制研究进展. 中国循证心血管医学杂志. 2020;12(01):126-8.

For peer review only - http://bmjopen.bmj.com/site/about/guidelines.xhtml

36. Gutiérrez E, Flammer AJ, Lerman LO, Elízaga J, Lerman A, Fernández-Avilés F. Endothelial dysfunction over the course of coronary artery disease. Eur Heart J. 2013;34(41):3175-81. Epub 20130907. doi: 10.1093/eurheartj/eht351. PubMed PMID: 24014385; PubMed Central PMCID: PMC3814514.
37. Yu S, Kim SR, Jiang K, Ogrodnik M, Zhu XY, Ferguson CM, et al. Quercetin Reverses Cardiac Systolic Dysfunction in Mice Fed with a High-Fat Diet: Role of Angiogenesis. Oxid Med Cell Longev. 2021;2021:8875729. Epub 20210219. doi: 10.1155/2021/8875729. PubMed PMID: 33688395; PubMed Central PMCID: PMC7914089.
38. Ziegler T, Abdel Rahman F, Jurisch V, Kupatt C. Atherosclerosis and the Capillary Network; Pathophysiology and Potential Therapeutic Strategies. Cells. 2019;9(1). Epub 20191224. doi: 10.3390/cells9010050. PubMed PMID: 31878229; PubMed Central PMCID: PMC7016600.
39. Reindl M, Reinstadler SJ, Feistritzer HJ, Theurl M, Basic D, Eigler C, et al. Relation of Low-Density Lipoprotein Cholesterol With Microvascular Injury and Clinical Outcome in Revascularized ST-Elevation Myocardial Infarction. J Am Heart Assoc. 2017;6(10). Epub 20171010. doi: 10.1161/jaha.117.006957. PubMed PMID: 29018020; PubMed Central PMCID: PMC5721881.
40. 2019 ESC/EAS guidelines for the management of dyslipidaemias: Lipid modification to reduce cardiovascular risk. Atherosclerosis. 2019;290:140-205. Epub 20190831. doi: 10.1016/j.atherosclerosis.2019.08.014. PubMed PMID: 31591002.
41. Baigent C, Blackwell L, Emberson J, Holland LE, Reith C, Bhala N, et al. Efficacy and safety of more intensive lowering of LDL cholesterol: a meta-analysis of data from 170,000 participants in 26 randomised trials. Lancet. 2010;376(9753):1670-81. Epub 20101108. doi: 10.1016/s0140-6736(10)61350-5. PubMed PMID: 21067804; PubMed Central PMCID: PMC2988224.
42. Zhang X, Hu D. [Comments for 2013 ACC/AHA Guideline on the Treatment of Blood Cholesterol to Reduce Atherosclerotic Cardiovascular Risk in Adults]. Zhonghua Nei Ke Za Zhi. 2015;54(1):9-12. PubMed PMID: 25877138.
43. Navarese EP, Robinson JG, Kowalewski M, Kolodziejczak M, Andreotti F, Bliden K, et al. Association Between Baseline LDL-C Level and Total and Cardiovascular Mortality After LDL-C Lowering: A Systematic Review and Meta-analysis. Jama. 2018;319(15):1566-79. doi: 10.1001/jama.2018.2525. PubMed PMID: 29677301; PubMed Central PMCID: PMC5933331.
44. Satny M, Hubacek JA, Vrablik M. Statins and Inflammation. Curr Atheroscler Rep. 2021;23(12):80. Epub 20211201. doi: 10.1007/s11883-021-00977-6. PubMed PMID: 34851454.
45. Ridker PM, Mora S, Rose L. Percent reduction in LDL cholesterol following high-intensity statin therapy: potential implications for guidelines and for the prescription of emerging lipid-lowering agents. Eur Heart J. 2016;37(17):1373-9. Epub 20160224. doi: 10.1093/eurheartj/ehw046. PubMed PMID: 26916794; PubMed Central PMCID: PMC4852064.
46. Kones R. Primary prevention of coronary heart disease: integration of new data, evolving views, revised goals, and role of rosuvastatin in management. A comprehensive survey. Drug Des Devel Ther. 2011;5:325-80. Epub 20110613. doi: 10.2147/dddt.S14934. PubMed PMID: 21792295; PubMed Central PMCID: PMC3140289.
47. Ragusa R, Basta G, Neglia D, De Caterina R, Del Turco S, Caselli C. PCSK9 and atherosclerosis: Looking beyond LDL regulation. Eur J Clin Invest. 2021;51(4):e13459. Epub 20201203. doi: 10.1111/eci.13459. PubMed PMID: 33236356.
48. Patriki D, Saravi SSS, Camici GG, Liberale L, Beer JH. PCSK 9: A Link Between Inflammation and Atherosclerosis. Curr Med Chem. 2022;29(2):251-67. doi: 10.2174/0929867328666210707192625.

For peer review only - http://bmjopen.bmj.com/site/about/guidelines.xhtml

PubMed PMID: 34238141.

49. Tavori H, Giunzioni I, Predazzi IM, Plubell D, Shivinsky A, Miles J, et al. Human PCSK9 promotes hepatic lipogenesis and atherosclerosis development via apoE- and LDLR-mediated mechanisms. Cardiovasc Res. 2016;110(2):268-78. Epub 20160315. doi: 10.1093/cvr/cvw053. PubMed PMID: 26980204; PubMed Central PMCID: PMC4836631.

50. Pęczek P, Leśniewski M, Mazurek T, Szarpak L, Filipiak KJ, Gąsecka A. Antiplatelet Effects of PCSK9 Inhibitors in Primary Hypercholesterolemia. Life (Basel). 2021;11(6). Epub 20210523. doi: 10.3390/life11060466. PubMed PMID: 34071103; PubMed Central PMCID: PMC8224623.

51. Ruscica M, Tokgözoğlu L, Corsini A, Sirtori CR. PCSK9 inhibition and inflammation: A narrative review. Atherosclerosis. 2019;288:146-55. Epub 20190717. doi: 10.1016/j.atherosclerosis.2019.07.015. PubMed PMID: 31404822.

52. 王晶，邓松柏，刘亚杰，佘强，杜建霖. PCSK9 抑制剂对极高危动脉粥样硬化性心血管疾病患者血脂谱的影响. 中华危重病急救医学. 2020;32(3):341-4. doi: 10.3760/cma.j.cn121430-20200129-00053.

53. Ridker PM, Bhatt DL, Pradhan AD, Glynn RJ, MacFadyen JG, Nissen SE. Inflammation and cholesterol as predictors of cardiovascular events among patients receiving statin therapy: a collaborative analysis of three randomised trials. Lancet. 2023. Epub 20230303. doi: 10.1016/s0140-6736(23)00215-5. PubMed PMID: 36893777.

54. 曹佳齐，郑宏超，丁跃有，周志文，赵莉芳. 增强型体外反搏治疗对非高危稳定型心绞痛患者疗效及心血管不良事件发生率的影响. 临床和实验医学杂志. 2020;19(05):500-2.

55. Schwartz GG, Steg PG, Szarek M, Bhatt DL, Bittner VA, Diaz R, et al. Alirocumab and Cardiovascular Outcomes after Acute Coronary Syndrome. N Engl J Med. 2018;379(22):2097-107. Epub 20181107. doi: 10.1056/NEJMoa1801174. PubMed PMID: 30403574.

56. Oyama K, Furtado RHM, Fagundes A, Jr., Zelniker TA, Tang M, Kuder J, et al. Effect of Evolocumab on Complex Coronary Disease Requiring Revascularization. J Am Coll Cardiol. 2021;77(3):259-67. Epub 20201113. doi: 10.1016/j.jacc.2020.11.011. PubMed PMID: 33197560.

57. Qian LJ, Gao Y, Zhang YM, Chu M, Yao J, Xu D. Therapeutic efficacy and safety of PCSK9-monoclonal antibodies on familial hypercholesterolemia and statin-intolerant patients: A meta-analysis of 15 randomized controlled trials. Sci Rep. 2017;7(1):238. Epub 20170322. doi: 10.1038/s41598-017-00316-3. PubMed PMID: 28331223; PubMed Central PMCID: PMC5428249.

For peer review only

For peer review only - http://bmjopen.bmj.com/site/about/guidelines.xhtml

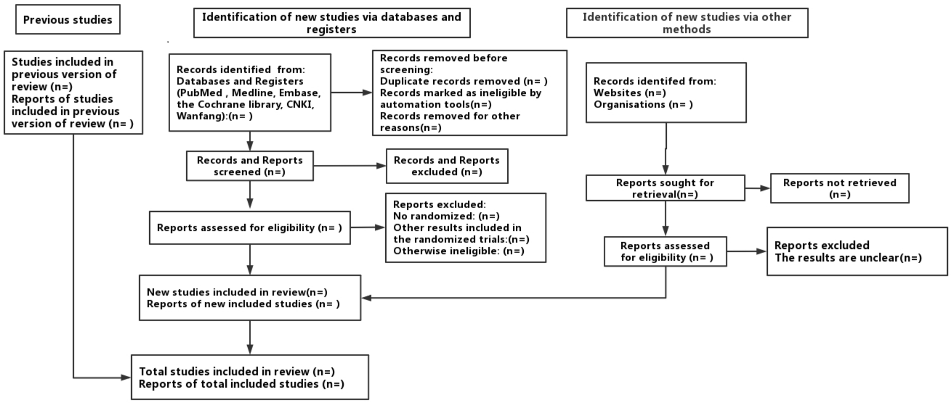

Fig 1 Flow Chart of literature Retrieval

116x52mm (300 x 300 DPI)

Table 1 Search strategy for PubMed

PCSK9 inhibitors [MeSH]
1. (proprotein convertase subtilisin/kexin type 9 monoclonal antibody) OR (PCSK9 inhibitor) OR (evolocumab) OR (alirocumab) OR (Praluent) OR (Repatha) [Title/Abstract]
Inflammation [MeSH]
2. (Inflammation) OR (Inflammatory response) OR (Inflammation reaction)
Randomized controlled trials [MeSH]
3. (RCT) OR (RCTs) OR (Randomized controlled trials) OR (Randomized controlled trial) [Title/Abstract]
Coronary heart disease[MeSH]
4. (Coronary artery disease) OR (Arteriosclerotic heart disease) OR (CAD) OR (CHD) OR (acute coronary syndrome) OR (chronic coronary syndrome) OR (ACS) OR (CCS)
5. 1 and 2 and 3 and 4

For peer review only

Table 2 Search strategy for EMBASE

PCSK9 inhibitors [MeSH]
1. (proprotein convertase subtilisin/kexin type 9 monoclonal antibody) OR (PCSK9 inhibitor) OR (evolocumab) OR (alirocumab) OR (Praluent) OR (Repatha) [Title/Abstract]

Inflammation [MeSH]
2. (Inflammation) OR (Inflammatory response) OR (Inflammation reaction)
Randomized controlled trials [MeSH]
3. (RCT) OR (RCTs) OR (Randomized controlled trials) OR (Randomized controlled trial) [Title/Abstract]
Coronary heart disease[MeSH]
4. (Coronary artery disease) OR (Arteriosclerotic heart disease) OR (CAD) OR (CHD) OR (acute coronary syndrome) OR (chronic coronary syndrome) OR (ACS) OR (CCS)
5. 1 and 2 and 3 and 4

Table 3 Search strategy for Web of Science

PCSK9 inhibitors [MeSH]
1. (proprotein convertase subtilisin/kexin type 9 monoclonal antibody) OR (PCSK9 inhibitor) OR (evolocumab) OR (alirocumab) OR (Praluent) OR (Repatha) [Title/Abstract]
Inflammation [MeSH]
2. (Inflammation) OR (Inflammatory response) OR (Inflammation reaction)
Randomized controlled trials [MeSH]
3. (RCT) OR (RCTs) OR (Randomized controlled trials) OR

For peer review only

(Randomized controlled trial) [Title/Abstract]
Coronary heart disease[MeSH]
4. (Coronary artery disease) OR (Arteriosclerotic heart disease) OR (CAD) OR (CHD) OR (acute coronary syndrome) OR (chronic coronary syndrome) OR (ACS) OR (CCS)
5. 1 and 2 and 3 and 4

For peer review only

Table 4 Search strategy for CNKI

PCSK9 抑制剂
1.(前蛋白转化酶枯草杆菌蛋白酶/可辛 9 型单克隆抗体) 或 (PCSK9 抑制剂) 或 (依洛尤单抗) 或 (阿利西尤单抗) 或 (波立达) 或 (瑞百安) [主题]
2. (炎症) 或 (炎症反应)
随机对照试验
3. (随机对照) 或 (随机对照试验) [主题]
冠状动脉粥样硬化性心脏病
4. (冠状动脉粥样硬化性心脏病) 或 (冠心病) 或 (急性冠脉综合征) 或 (慢性冠脉综合征) 或 (急性心肌梗死) 或 (ST 段抬高型心肌梗死) 或 (心绞痛) 或 (稳定性心绞痛) 或 (不稳定性心绞痛)
5. 1 和 2 和 3 和 4

Table 5 Search strategy for Wanfang

PCSK9 抑制剂
1.(前蛋白转化酶枯草杆菌蛋白酶/可辛 9 型单克隆抗体) 或 (PCSK9 抑制剂) 或 (依洛尤单抗) 或 (阿利西尤单抗) 或 (波立达) 或 (瑞百安) [主题]
2. (炎症) 或 (炎症反应)
随机对照试验
3. (随机对照) 或 (随机对照试验) [主题]
冠状动脉粥样硬化性心脏病
4. (冠状动脉粥样硬化性心脏病) 或 (冠心病) 或 (急性冠脉综合征) 或 (慢性冠脉综合征) 或 (急性心肌梗死) 或 (ST 段抬高型心肌梗死) 或 (心绞痛) 或 (稳定性心绞痛) 或 (不稳定性心绞痛)
5. 1 和 2 和 3 和 4

Table 6 Search strategy for VIP database

PCSK9 抑制剂
1.(前蛋白转化酶枯草杆菌蛋白酶/可辛 9 型单克隆抗体) 或 (PCSK9 抑制剂) 或 (依洛尤单抗) 或 (阿利西尤单抗) 或 (波立达) 或 (瑞百安) [主题]
2. (炎症) 或 (炎症反应)
随机对照试验
3. (随机对照) 或 (随机对照试验) [主题]

For peer review only

冠状动脉粥样硬化性心脏病
4. (冠状动脉粥样硬化性心脏病) 或 (冠心病) 或 (急性冠脉综合征) 或 (慢性冠脉综合征) 或 (急性心肌梗死) 或 (ST 段抬高型心肌梗死) 或 (心绞痛) 或 (稳定性心绞痛) 或 (不稳定性心绞痛)
5. 1 和 2 和 3 和 4

For peer review only

Reporting checklist for protocol of a systematic review and meta analysis.

Based on the PRISMA-P guidelines.

Instructions to authors

Complete this checklist by entering the page numbers from your manuscript where readers will find each of the items listed below.

Your article may not currently address all the items on the checklist. Please modify your text to include the missing information. If you are certain that an item does not apply, please write "n/a" and provide a short explanation.

Upload your completed checklist as an extra file when you submit to a journal.

In your methods section, say that you used the PRISMA-Preporting guidelines, and cite them as:

Moher D, Shamseer L, Clarke M, Ghersi D, Liberati A, Petticrew M, Shekelle P, Stewart LA. Preferred Reporting Items for Systematic Review and Meta-Analysis Protocols (PRISMA-P) 2015 statement. Syst Rev. 2015;4(1):1.

		Reporting Item	Page Number
**Title**			
Identification	#1a	Identify the report as a protocol of a systematic review	1
Update	#1b	If the protocol is for an update of a previous systematic review, identify as such	1
**Registration**			
	#2	If registered, provide the name of the registry (such as PROSPERO) and registration number	2
**Authors**			
Contact	#3a	Provide name, institutional affiliation, e-mail address of all protocol authors; provide physical mailing address of corresponding author	1
Contribution	#3b	Describe contributions of protocol authors and identify the guarantor of the review	20

For peer review only

**Amendments**			
	#4	If the protocol represents an amendment of a previously completed or published protocol, identify as such and list changes; otherwise, state plan for documenting important protocol amendments	n/a,This item is not apply to the protocol.
**Support**			
Sources	#5a	Indicate sources of financial or other support for the review	20
Sponsor	#5b	Provide name for the review funder and / or sponsor	20
Role of sponsor or funder	#5c	Describe roles of funder(s), sponsor(s), and / or institution(s), if any, in developing the protocol	20
**Introduction**			
Rationale	#6	Describe the rationale for the review in the context of what is already known	3-6
Objectives	#7	Provide an explicit statement of the question(s) the review will address with reference to participants, interventions, comparators, and outcomes (PICO)	3-6
**Methods**			
Eligibility criteria	#8	Specify the study characteristics (such as PICO, study design, setting, time frame) and report characteristics (such as years considered, language, publication status) to be used as criteria for eligibility for the review	11-12
Information sources	#9	Describe all intended information sources (such as electronic databases, contact with study authors, trial registers or other grey literature sources) with planned dates of coverage	6-9
Search strategy	#10	Present draft of search strategy to be used for at least one electronic database, including planned limits, such that it could be repeated	8-9
Study records - data management	#11a	Describe the mechanism(s) that will be used to manage records and data throughout the review	7

For peer review only

Study records - selection process	#11b	State the process that will be used for selecting studies (such as two independent reviewers) through each phase of the review (that is, screening, eligibility and inclusion in meta-analysis)	9-11
Study records - data collection process	#11c	Describe planned method of extracting data from reports (such as piloting forms, done independently, in duplicate), any processes for obtaining and confirming data from investigators	11-13
Data items	#12	List and define all variables for which data will be sought (such as PICO items, funding sources), any pre-planned data assumptions and simplifications	11-12
Outcomes and prioritization	#13	List and define all outcomes for which data will be sought, including prioritization of main and additional outcomes, with rationale	12
Risk of bias in individual studies	#14	Describe anticipated methods for assessing risk of bias of individual studies, including whether this will be done at the outcome or study level, or both; state how this information will be used in data synthesis	12-14
Data synthesis	#15a	Describe criteria under which study data will be quantitatively synthesised	12-13
Data synthesis	#15b	If data are appropriate for quantitative synthesis, describe planned summary measures, methods of handling data and methods of combining data from studies, including any planned exploration of consistency (such as I2, Kendall's τ)	12-16
Data synthesis	#15c	Describe any proposed additional analyses (such as sensitivity or subgroup analyses, meta-regression)	15
Data synthesis	#15d	If quantitative synthesis is not appropriate, describe the type of summary planned	12-13
Meta-bias(es)	#16	Specify any planned assessment of meta-bias(es) (such as publication bias across studies, selective reporting within studies)	14
Confidence in cumulative	#17	Describe how the strength of the body of evidence will be assessed (such as GRADE)	15-16

For peer review only

evidence

Notes:

- 4: n/a,This item is not apply to the protocol. The PRISMA-P elaboration and explanation paper is distributed under the terms of the Creative Commons Attribution License CC-BY. This checklist was completed on 26. March 2023 using https://www.goodreports.org/, a tool made by the EQUATOR Network in collaboration with Penelope.ai

For peer review only
